# Domain-adaptive Bengali text summarization: Harnessing transformer-based large language models in closed-set contexts

**DOI:** 10.1371/journal.pone.0357890

**Published:** 2026-10-05

**Authors:** Anusree Roy, Fahrin Hossain Sunaira, Tateyama Orpa, Farasha Shamma Yussouf, Intisar Tahmid Naheen, Riasat Khan

**Affiliations:** Electrical and Computer Engineering, North South University, Dhaka, Bangladesh; Hunan Normal University, CHINA

## Abstract

This work aims to develop a domain-adapted Bengali text summarization model by training and fine-tuning on general and domain-specific datasets with categories such as state, international, and sports. Flan-T5 and mT5 LLMs were trained on the XLSUM Bengali dataset for text summarization, and they were trained on source domains and fine-tuned on the target domains for domain adaptation. In general-domain summarization on the XLSum Bengali dataset, mT5 achieved stronger overall performance than Flan-T5, with ROUGE-1 and BERTScore values of 0.21 and 0.72, respectively. The Flan-T5-XLSUM model outperformed other LLMs, achieving a ROUGE score of 0.79, a BLEU score of 0.13 and a BERTScore of 0.86. A human evaluation involving 28 participants was conducted to assess summary fluency, adequacy, and factual consistency across domains, confirming the qualitative effectiveness of the proposed models. A custom dataset is developed with manually annotated categories for domain adaptation purposes, contributing to domain-specific Bengali data. The ablation study illustrated that pre-training and fine-tuning on domain-specific data significantly enhanced model performance, with Flan-T5 fine-tuned on sports data. Three explainability methods (LIME, SHAP, and BertViz) revealed that geographically and contextually meaningful tokens strongly influenced the domain-adaptive Flan-T5 Bengali summarization model. The adapter-based Flan-T5 architecture enables effective multi-domain fine-tuning with less than 1% of trainable parameters while preserving model performance.

## Introduction

Text summarization is the procedure of shortening a large body of text into a condensed form while keeping the essential information within the overall text [1]. This approach is crucial in various aspects, including news reporting, where it helps readers quickly grasp the key points of the lengthy articles. Considering Bengali is spoken by over 250 million people worldwide [2], an efficient text summarization tool is essential, especially for news consumption. Summarizing Bengali news articles can significantly improve the accessibility and distribution of information, enabling readers to stay informed without needing to look through lengthy content [3]. Text summarization is vital in Natural Language Processing (NLP), where the objective is to condense long texts into shorter and informative summaries. There is a noticeable lack of comprehensive datasets for the Bengali language. Few works [4,5] on Bengali text summarization utilize the same few public datasets that are available. The absence of such datasets hampers the development and refinement of summarization techniques tailored for Bengali text summarization [6].

Recent studies show the significant advancements offered by Large Language Models (LLMs) in various natural language processing tasks, including abstractive text summarization. Research has examined the domain adaptation potential of LLMs for summarization tasks, analyzing how these models perform when adapted to specific domains [7].

Domain adaptation is a specific case of transfer learning in which the task remains the same, but the domains differ. It aims to improve model performance on a target domain with insufficient annotated data by leveraging knowledge from a related source domain with sufficient labeled data. Unlike general transfer learning, which may involve changes in both tasks and domains, domain adaptation focuses on aligning the distributions between source and target domains to enhance model effectiveness. This approach enables models to adapt to new domains with limited annotated data by transferring knowledge from related domains [8]. Domain adaptation is a critical approach for enhancing summarization quality. It involves fine-tuning a model on a specific domain’s data, which allows the model to better understand domain-specific terminology and context. This process enables models to more accurately capture the vocabulary, structure, and contextual nuances unique to particular domains, such as news, healthcare, or finance [9]. Significant progress in LLMs has been documented across various natural language processing tasks, including abstractive text summarization, which entails generating a condensed version of the most relevant content from a document [10]. While research on domain adaptation in LLMs for summarization has increased, most studies have focused on single domains, such as news articles or clinical reports. Research is needed across multiple domains to better understand how these models adapt to different target areas.

When LLMs are applied to specific domains, they require corresponding domain-specific knowledge bases for effective training. This requirement leads to a limitation wherein a model trained in one domain struggles to generalize to others, resulting in resource inefficiency. The issue primarily stems from the disparity between the data distributions of the training and target domains. This challenge becomes particularly significant when dealing with specialized topics such as sports, international affairs, or state-related content, where general-purpose models may fail to generate relevant summaries. Developing methods to bridge this gap and improve the model’s adaptability and efficiency in summarization tasks is essential. Domain adaptation aims to train models using multiple source domains to enable generalization to new, unseen domains. Consequently, enhancing domain adaptation performance is a critical objective for large-scale models to improve their effectiveness in downstream tasks. Investigating the factors that influence domain adaptation performance remains an important area of focus [9].

In response to the challenges in low-resource domain scenarios, particularly for abstractive summarization, domain adaptation methods have emerged as vital solutions. These approaches enable models to quickly adapt to target domain tasks despite the scarcity of labeled data. Only a limited number of studies have applied domain adaptation techniques to low-resource scenarios in the context of abstractive summarization. Such methods simulate low-resource conditions by reducing the number of training samples across various domains, evaluating the adaptability and performance of summarization models under constrained settings [11].

The concept of domain adaptation and fine-tuning-based training for abstractive text summarization can be explored under various scenarios, including monolingual, cross-lingual, and multilingual tasks. In domain adaptation, fine-tuning tends to yield better results when sufficient training data is available and is particularly effective when there are significant differences between the source and target domains [12]. This approach is employed in the present research.

Some research gaps remain in domain-specific abstractive text summarization. For instance, handling long input texts remains a challenge, necessitating the use of efficient transformer architectures. To ensure good accuracy, it is suggested to apply advanced evaluation metrics such as BERTScore, etc., so that we can easily detect and fix model hallucinations. Lastly, the problems with models being trained on general-purpose data have been addressed as they perform poorly with specialized content because they lack domain-specific knowledge. Fine-tuning pre-trained models on domain-specific data offers a potential solution to address this issue.

This research implements a domain-adaptive approach for Bengali text summarization and brings forth the following contributions:

- A significant contribution has been made by developing a novel Bengali text summarization model through the fine-tuning of LLMs, mT5 and Flan-T5. It addresses the lack of effective summarization models for Bengali, a low-resource language, improving accessibility and usability.
- A custom dataset comprising 21,512 samples is introduced, created by scraping Bengali news articles from various newspapers. Each entry has been manually categorized into seven domains: sports, states, entertainment, international, education, economy, and technology. This structured dataset enables targeted text summarization across various fields.
- The LLMs are fine-tuned across three major domains – state, international, and sports, which are selected based on the highest number of dataset entries. This domain-specific training enhances the quality and relevance of the generated summaries.
- The adaptability of LLMs in low-resource settings is demonstrated by employing a structured domain-based approach. The results indicate that domain adaptation techniques can significantly improve the effectiveness of Bengali text summarization.
- Ablation studies are conducted, demonstrating that combining pre-training with domain-specific fine-tuning significantly enhances Bengali text summarization performance, with Flan-T5 achieving the best results.
- This study introduces an adapter-based domain adaptation approach that requires tuning less than 1% of model parameters, which provides computational efficiency while improving domain alignment and generation quality.
- An external evaluation involving 28 participants was conducted, and the generated summaries were assessed for fluency, adequacy, and consistency, providing qualitative insights that complemented the automatic metrics.

The novelty of this work is implementing a domain-adaptive Bengali text summarization framework using fine-tuned large language models (mT5 and Flan-T5) on a newly created, manually categorized dataset, demonstrating improved summarization performance in low-resource, domain-specific contexts.

## Literature review

### Text summarization using large language models

Basyal et al. [13] implemented text summarization with a diverse set of LLMs. The authors investigated the performance of various LLMs, e.g., MPT-7B-Instruct, Falcon-7B-Instruct, and OpenAI ChatGPT textdavinci-003, when applied to different datasets, i.e., XSUM and CNN-DailyMail News Text Summarization datasets. From their experiment, text-davinci-003 outperformed the others on both datasets. Borah et al. [14] explored the T5 model’s effectiveness in abstractive text summarization on datasets, e.g., CNNDM, MSMO, and XSUM. Their ROUGE and BLEU scores demonstrated the model’s superior capability in generating concise summaries, particularly excelling with the MSMO dataset. Lewis et al. [15] introduced the BART model, which outperformed existing models in text summarization tasks, achieving top ROUGE scores of 44.16 on the dataset CNN/DailyMail and 45.14 on XSum. Song et al. [16] employed FineSurE, a novel evaluation framework that employed LLMs to assess summarization quality across linguistic, content, and factual dimensions. The system outperformed current approaches in both general and domain-specific summarization tasks, achieving state-of-the-art accuracy with correlations as high as 0.92 with human assessments on benchmark datasets. Kolagar et al. [17] focused on uncertainty propagation in text summarization using LLMs, including GPT-4 and T5. The authors implemented model-based uncertainty metrics in their framework to quantify and align uncertainty transfer. Demonstrated on benchmark datasets, such as CNN/DailyMail and XSum, their method significantly reduced uncertainty mismatch, with alignment measures demonstrating improvements of up to 15% above baselines.

### Text summarization for Bengali texts

Shahariar et al. [4] introduced an effective approach where a single text was first summarized using four pre-trained Bengali text summarization models. Its respective human-written reference summary was considered. The top-ranked summary was selected based on the summary similarity assessment, summary similarity matrix calculation, graph building, and ranking. The mT5 XLSum model performed better than other models on the Bengali Text Summarization dataset. Kowsher et al. [18] introduced a monolingual BERT model for the Bengali language called Bangla-BERT. The authors constructed a Bengali language model dataset, BanglaLM. They addressed the mBERT framework’s limitation for Bengali trained on limited and more structured data and mixed weights issues among 104 languages. The fine-tuned technique outperformed other prior state-of-the-art models. Bhattacharjee et al. [5] developed a seq2seq LSTM model with a local attention mechanism for Bengali abstractive news summarization, which effectively generated coherent and contextually rich summaries from a large dataset of over 19,000 articles. Chowdhury et al. [19] developed a graph-based unsupervised approach that leveraged the seq2seq architecture to enhance the abstractive summarization of Bengali texts, which were notably low-resource, demonstrating significant potential for natural language processing applications in under-resourced languages. Jahan et.al [20] developed a unique lexicon-based technique for Bengali text summarization, which effectively summarized text and extracted meaningful sentences using rule-based algorithms and a sentiment lexicon. When assessed on a dataset of Bengali text, the method’s summarization accuracy was 78%, indicating that it was capable of generating meaningful and eloquent summaries without the need for machine learning models.

### Bengali text summarization using LLMs

Rony et al. [21] evaluated the performance of LLMs, including GPT-4 and mT5, for Bengali text summarization. Using Bengali-specific datasets, the applied GPT-4 outperformed other models, achieving the highest ROUGE-L score of 82%. The results illustrated how well LLMs could handle low-resource languages, such as Bengali, but they stressed the crucial role of dataset quality and fine-tuning in achieving the best results. Lora et al. [22] explored ConVerSum, a framework that utilized contrastive learning for cross-lingual summarization for Bengali text summarization. To align semantically similar words across languages, the technique incorporated pre-trained models, mBART and XLM-R for representation learning in conjunction with a contrastive loss. ConVerSum outperformed current techniques in terms of accuracy and fluency, especially in situations where data was scarce, with a ROUGE-L score of 84% for Bengali cross-lingual summary tasks when analyzed on benchmark datasets. Parnell et al. [23] introduced a differentiable pipeline designed for few-shot cross-lingual summarization. Effective summarization in low-resource languages was made possible by the framework’s combination of few-shot learning with pre-trained models, T5 and mBART. With a ROUGE-L score of 85% for Bengali text summarization, the method showed promise for scalable cross-lingual applications and performed well even with minimal training data. Another work in the field of Bengali text summarization using LLMs is conducted by Raihan et al. [24], where they used Tiger LLM, a variant of the LLaMA model fine-tuned specifically for the Bengali language. The model’s abilities were demonstrated in various NLP tasks, such as summarizing Bengali text. The model outperformed other LLMs, such as mBERT and various conventional methods, achieving a ROUGE-L score of 88% on Bengali text summarizing tasks.

### Domain adaptation

Busto et al. [25] explored the problem of domain adaptation in scenarios where the target domain contained categories not present in the source domain. This approach, known as Open Set Domain Adaptation (OSDA), was particularly valuable in real-world applications where data in the target domain included instances of unknown classes that were not in the source domain. The authors conducted extensive experiments on datasets including the Office dataset (images of office supplies), the Caltech dataset, and video datasets for action recognition. Their model, referred to as ATI-λ (Assign-and-Transform-Iteratively with Outlier handling), showed strong performance on open-set tasks by accurately identifying unknown categories in the target domain. Cao and his team [26] addressed the limitations of conventional domain adaptation approaches when dealing with partial transfer learning techniques. In these scenarios, only a subset of classes from the source domain overlapped with the target domain, making it essential to ignore irrelevant source classes to avoid negative transfer. The model was evaluated on datasets such as, Office-31, Caltech-Office, and ImageNet-Caltech, covering diverse transfer learning scenarios. The authors introduced the Selective Adversarial Network (SAN), which selectively aligned the source and target domains by focusing only on shared classes while disregarding irrelevant source classes. You et al. [27] developed a new approach to domain adaptation called Universal Domain Adaptation (UDA). This framework is designed for scenarios where the relationship between the label sets of the source and target domains is unknown, enabling the model to handle a mix of shared and private classes in both domains.

### Domain adaptation for text summarization

Afzal et al. [7] explored the domain adaptation capabilities of various large language models in text summarization. It evaluated multiple models, including conventional encoder-decoder models and LLMs of different sizes, across three domains: scientific, medical, and governmental. The evaluation was conducted using two methods: fine-tuning and in-context learning (ICL). The authors presented a domain adaptation evaluation suite called AdaptEval, which included a domain-specific benchmark and metrics such as ROUGE, BERTScore, domain vocabulary overlap (DVO), token distribution shift, and G-eval. The results showed that LLMs performed comparably well in the ICL setting, and fine-tuning models achieved better automatic evaluation scores. Lu and colleagues [28] explored various strategies for fine-tuning large language models to enhance their domain adaptation capabilities. They examined how different fine-tuning techniques, such as CPT and SFT, impacted LLM performance in specialized domains. Results revealed that models fine-tuned and merged using SLERP and preference optimization strategies achieved higher accuracy in domain-specific tasks. Zhong et al. [29] introduced MTL-DAS, a model for automatic text summarization focused on domain adaptation, by using transformer-based models that had already been trained, such as BERT, and fine-tuning them for particular domains. To improve summarizing performance, the authors provided a multi-task learning (MTL) approach that used domain-specific data to modify summarization models for specific domains. When tested on domain-specific datasets, MTL-DAS outperformed conventional summarization models and significantly improved ROUGE-L scores, with the highest result being approximately 90%. Other significant approaches in the field of domain adaptation for text summarization included the study by Mao et al. [30], where they introduced CiteSum, a framework for scientific extreme summarization. This framework used citation text to direct the summarizing process and could adapt to certain domains with minimal supervision. The authors utilized a small set of labeled data from scientific citation datasets, including publications from repositories, arXiv and PubMed, to fine-tune transformer-based models, BERT and T5. CiteSum performed impressively with ROUGE-L score reaching up to 90% proving its capacity to produce concise and insightful summaries while maintaining accuracy in domain adaptation. The Kernelized Unified Framework for Domain Adaptation (KUFDA), which was developed by Sanodiya et al. [31] uses a kernelized approach that aligns source and target domains geometrically and statistically in tandem. KUFDA was able to jointly minimize divergence in marginal and conditional distributions via Maximum Mean Discrepancy (MMD) and uphold discriminative and manifold structure, resulting in mean accuracies of 86.83% on the Office-Caltech dataset and 74.42% on the PIE Face dataset; these results surpassed those of many of the state-of-the-art methods at the time. Paul et al. [32] proposed 3-GANSumm, an unsupervised domain-adaptive summarization framework based on a Triple-GAN and graph attention networks for dynamically clustering and summarizing microblogging data streams during disaster events. The experiments demonstrated that 3-GANSumm outperformed traditional extractive summarization methods such as LexRank and LSA in ROUGE-L F-measure scores, demonstrating the benefit of GAN-based approaches to real-time and low-resource summarization tasks, including applications to Bangla.

Antonio et al. [33] investigated graph-based text modeling approaches employing complex-network analysis of word usage consistency and forma mentis networks for text representation. The authors provided valuable perspectives for identifying important textual structures and central sentences. Stell et al. [34] forma mentis networks for neural summarization. Sentence or word centrality scores as auxiliary features, ranking signals, or pre-selection mechanisms are applied before abstractive summary generation. The authors proved that graph-based representations could be combined with the domain-adapted frameworks.

A comparative analysis of some of the related works on Bengali Text Summarization using LLMs and Domain Adaptation provided in the literature review section is summarized in Table 1.

**Table 1:** Summary of related studies on Bengali and multilingual text summarization models.

Ref.	Subject	Dataset used and size	Model used and evaluation metrics	Advantages	Limitations
[13]	Text summarization using different LLM models	CNN/DailyMail 3.0.0 and XSum datasets (201k training samples)	mpt-7b-instruct, falcon-7b-instruct, text-davinci-003; evaluated with BLEU, ROUGE-1/2/L, BERTScore	Large models fine-tuned on domains	Evaluation limited to two datasets
[14]	Comparative analysis of T5 for summarization	CNN/DM, MSMO, XSum datasets	T5 model; evaluated using ROUGE, BLEU	Adaptable for multiple NLP tasks	High computational demands, inconsistent summaries
[4]	Bengali text summarization via ranking	XL-Sum (10,126), Bangla Text Summ. (80.3k)	mT5-XL, CrossSum, scibert, mT5; evaluated with BLEU, ROUGE, BERTScore, WIL, METEOR	Multi-model comparison for Bangla	Inconsistent outputs across datasets
[18]	Bangla-BERT: monolingual BERT model for Bengali	BanglaLM dataset	Bangla-BERT (Transformer-based); evaluated with acc., precision, recall, F1, AUC	Better than mBERT on Bangla NLP tasks	High training/inference cost; needs large domain-specific data
[21]	Summarization of Bangla texts using LLMs	BANS and BNLPC datasets, 50 validation samples for human eval.	GPT-3.5, GPT-4, OPT, LLaMA2, PaLM-2; BLEURT, ROUGE, BERTScore, BARTScore, METEOR	GPT-4 outperformed others	Small sample size for human evaluation
[7]	Domain-wise LLM summarization eval.	PubMed (215.8k), GovReport (193k)	GPT-4, ChatGPT, Falcon, LLaMA2, Vicuna, Mistral, PEGASUS; ROUGE, BERTScore, DVO	Multi-domain performance tested	Limited GPT-4 samples due to cost
[15]	BART: Denoising sequence-to-sequence pretraining	GLUE, SQuAD 1.1/2.0, CNN/DM, XSum, MNLI, WMT16	BART; ROUGE, BLEU, PPL, F1, Accuracy, MCC, MET	Combines bidirectional enc. + autoregressive dec.	High cost, dataset dependence, less effective on loosely aligned tasks
[5]	Bengali abstractive summarization with attention	BANPS corpus (19,096 articles)	LSTM-based model; ROUGE, BLEU, user ratings	Human-like fluent summaries	Struggled with long words, truncation, repetition
[23]	SUMTRA: Few-shot cross-lingual summarization	CrossSum, WikiLingua datasets (varied by language)	SUMTRA (mBART-50 variants); evaluated with mROUGE, BERTScore	Modular, few-shot, good for low-resource languages	Requires high-quality summarization + translation modules; English-to-many only
[26]	Partial transfer learning via selective adversarial networks	Office-31 (4,652 images), Caltech-Office, ImageNet-Caltech	SAN (Selective Adversarial Networks); classification accuracy	Avoids negative transfer, state-of-the-art performance	High cost, needs reliable target data probabilities

## Methodology

### Problem formulation

Fig 1 shows the primary steps of the proposed Bengali text summarization system. In order to formally outline the methodology, let $X_{i}$ represent the input Bengali text sequence, and *i* stand for the sample index. A tokenized sequence $T_{i}$ of fixed length *L* is generated from $X_{i}$ following pre-processing (including normalization, tokenization, and padding). In order to preserve sequence order, positional encodings $P_{i}$ are added to the dense vector $E_{i}\in {\mathbb{R}}^{d}$, where *d* is the embedding dimension. Semantic latent representations $h_{i}\in {\mathbb{R}}^{m}$ are obtained by passing the combined representation $E_{i}+P_{i}$ through the encoder. The tokenized summary ${\hat{Y}}_{i}=\{{\hat{y}}_{1},{\hat{y}}_{2},...,{\hat{y}}_{L}\}$ is predicted by the decoder leveraging the latent representations $h_{i}$, where ${\hat{y}}_{k}$ denotes the *k* -th token in the predicted summary. For domain adaptation, the predicted domain label ${\hat{Y}}_{\text{domain}}$ is compared to the ground truth domain label *Y*_domain_ in order to compute the domain classification loss $L_{D}$ using the latent representation $h_{i}$. To determine the domain confusion loss $L_{DC}$, the gradient reversal layer transforms $h_{i}$ to create domain-invariant embeddings ${\tilde{h}}_{i}\in {\mathbb{R}}^{m}$. These embeddings have been configured for maintaining task-relevant information while minimizing domain-specific unpredictability. The total loss function is given as:


$$\begin{array}{c} L_{\text{total}}=L_{\text{CE}}+\lambda_{D}L_{D}+\lambda_{DC}L_{DC} \end{array}$$ (1)

**Fig 1:**
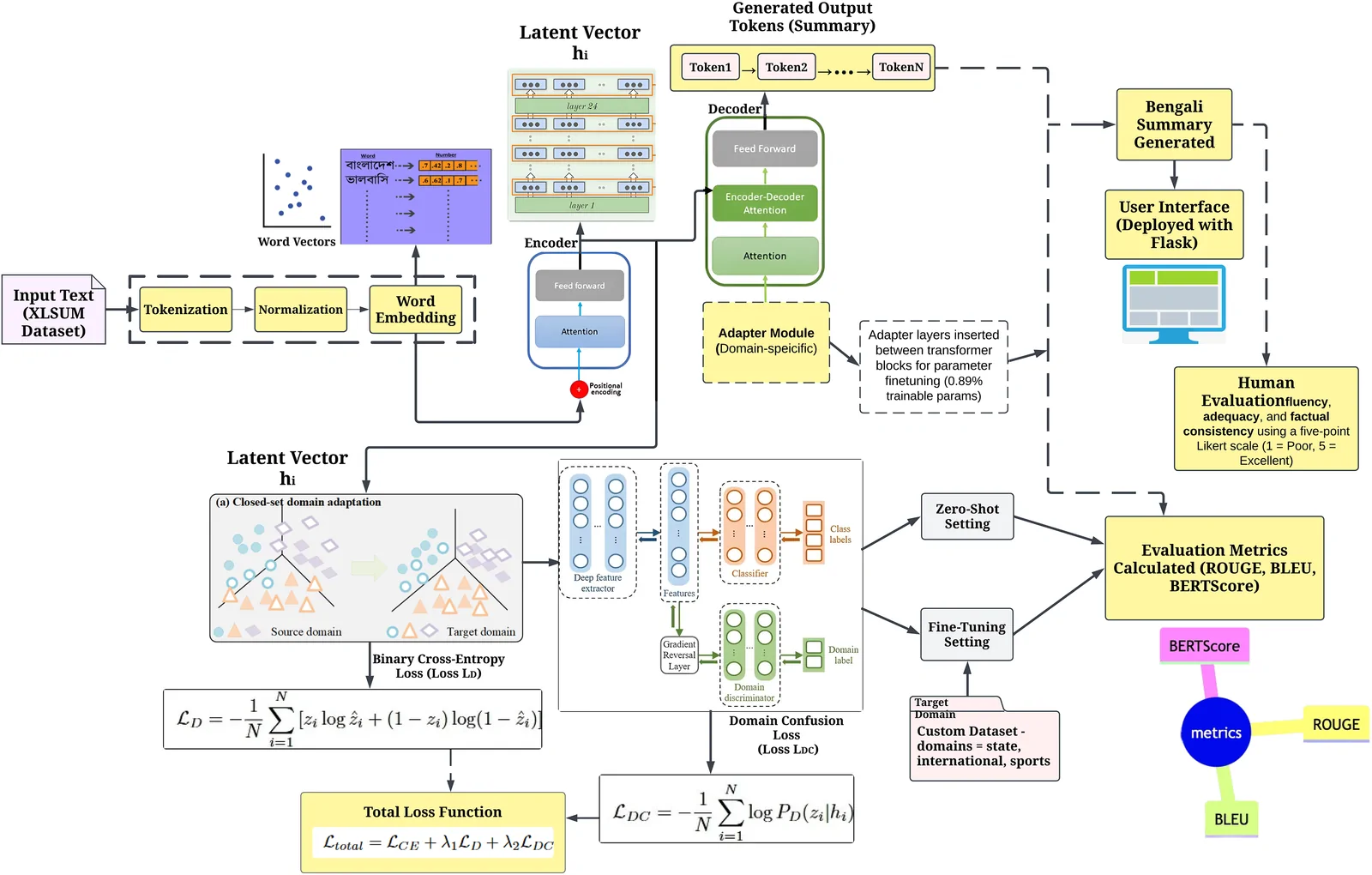
Proposed workflow for closed-set domain-adaptive Bangla text summarization with traditional fine-tuning, parameter-efficient fine-tuning, and hybrid evaluation.

where the error between the ground truth $Y_{i}$ and the predicted summary ${\hat{Y}}_{i}$ is evaluated by *L*_CE_. The contributions of the domain adaptation losses are balanced by the scaling factors $\lambda_{D}$ and $\lambda_{DC}$. The same pipeline is implemented to process the test and validation inputs $X_{i}$ during the evaluation process. As illustrated in Fig 1, the proposed adapter-augmented Flan-T5 architecture integrates lightweight domain-specific adapter modules between the encoder and decoder transformer layers, enabling parameter-efficient fine-tuning while preserving the core pre-trained model parameters. Performance metrics, including BERTScore, ROUGE, and BLEU, are applied to assess the quality of the predicted summaries. Before deployment, the final output ${\hat{Y}}_{i}$ may potentially require post-processing.

### Custom dataset preparation

For domain adaptation, a dataset comprising 21,512 rows was created by web scraping news articles from various popular online newspapers of Bangladesh, including “The Daily Star”, “Prothom Alo”, “Jugantor” and “Ittefaq.” The web scraping process was conducted using Python’s Beautiful Soup library, a Python package designed to parse HTML and XML documents. Beautiful Soup generates a parse tree from HTML content, enabling efficient data extraction required for web scraping tasks. In the Python code, class names of HTML elements—specifically <h1> and <p> tags—were identified and used to extract data based on their associated class names. Since different online newspapers employed different class name structures, the code was adapted accordingly for each source. The title of each news article was labeled under the ‘title’ column, the headline at the top of the article, containing summarized information, was labeled as the ‘summary’ column, and the full body text describing the news was labeled as the ‘text’ column. A total of 22,100 rows were collected. The dataset was manually annotated and categorized into seven distinct domains: sports, international, state, entertainment, economy, education, and technology. No login credentials, personally identifiable information, or restricted materials were accessed during data collection. The data collection process complied with the Terms of Use of each source website, which permit access to and use of publicly accessible content for non-commercial academic research. The dataset used in this study, titled “BanglaSumXL Categories,” is publicly available on the Hugging Face Datasets platform at: https://huggingface.co/datasets/midnightGlow/BanglaSUM_Eng.

#### Manual annotation process.

The annotation process was conducted by a team of four annotators. All annotators were female, within the age range of 23–24 years. Each annotator was a university student with an academic background in Computer Science and Engineering. The details of the annotators are given in Table 2.

**Table 2:** Annotator details of the proposed Bangla text summarization research.

Attribute	Value
Number of Annotators	4
Gender Distribution	4 female
Age Range	23–24 years
Educational Background	All annotators were university students with a background in Computer Science and Engineering.

The annotators individually labeled each of the articles. Disagreements during the annotation process were resolved through mutual discussion among the annotators. In cases where consensus could not be achieved, the final label was determined based on a majority vote.

#### Word and token distribution.

The average length of articles and summaries, measured in words, for each category is summarized in Table 3.

**Table 3:** Average article and summary lengths by category of the custom dataset.

Category	Avg. Article Length (Words)	Avg. Summary Length (Words)
Economy	563.65	33.91
Education	476.43	39.98
Entertainment	316.51	35.48
International	462.72	39.93
Sports	377.94	35.15
State	406.76	39.71
Technology	541.15	30.87

The average article length varies across categories. The Economy and Technology categories contain the longest articles (approximately 563 and 541 words, respectively), whereas Entertainment articles are the shortest, averaging around 316 words. The length of summaries remains relatively consistent across categories, ranging between 30 and 40 words. This indicates that articles in the Economy and Technology categories are more detailed, while those in Entertainment and Sports are comparatively concise. Following the annotation, duplicates and null values were removed from the dataset. After performing Exploratory Data Analysis (EDA), the dataset was refined to 21,512 rows. Fig 2 illustrates a sample from the custom dataset, which contains four columns: category, title, text, and summary.

**Fig 2:**
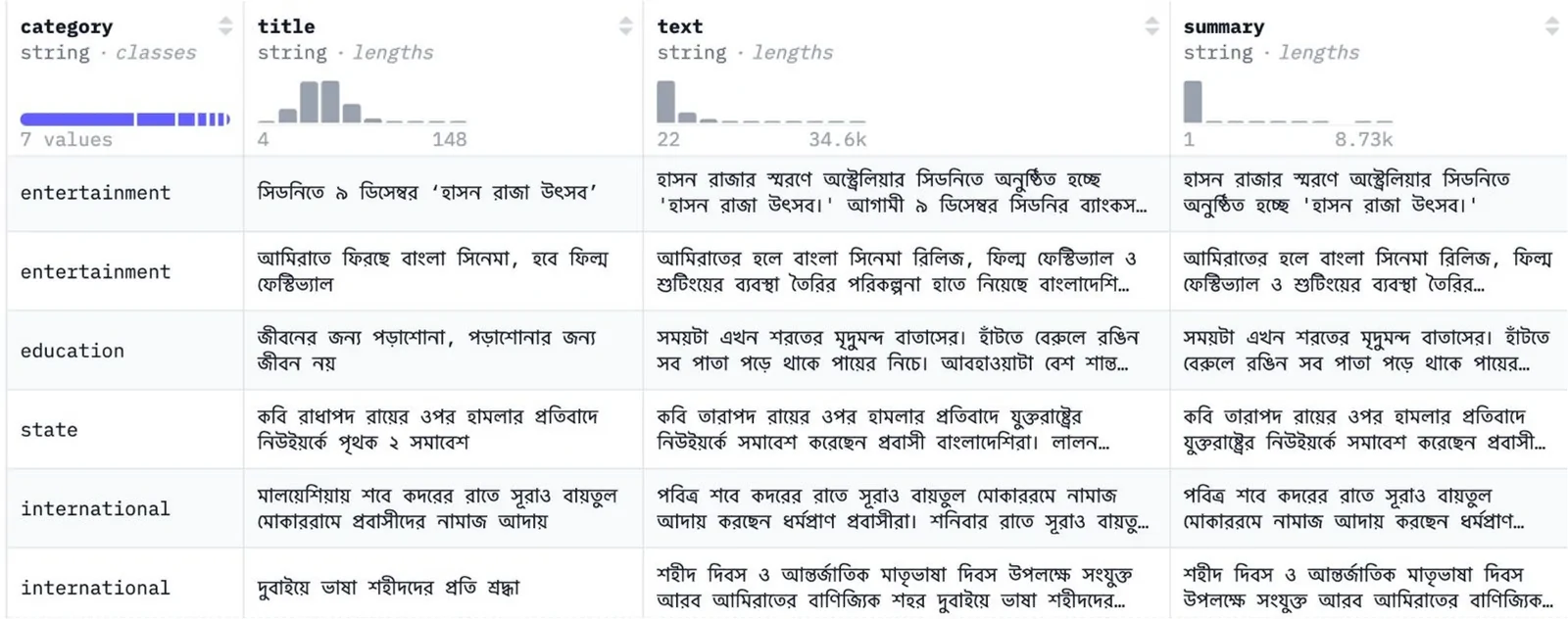
Sample entries from the custom Bengali news summarization dataset.

For EDA, Python’s Pandas library was used as the primary data manipulation tool. After removing duplicates and null values, data sampling was conducted for the domain adaptation phase due to the state category having a disproportionately large number of entries compared to other categories in both the custom (target domain) and source domain datasets. To perform resampling, the resample function from the scikit-learn library was employed. The resampled datasets for both source and target domains were obtained with the following distribution: 42.9% for state, 35.7% for international, and 21.4% for sports, as illustrated in Fig 3. English translations of the Bengali text shown in this figure is provided in Supplementary S1 File.

**Fig 3:**
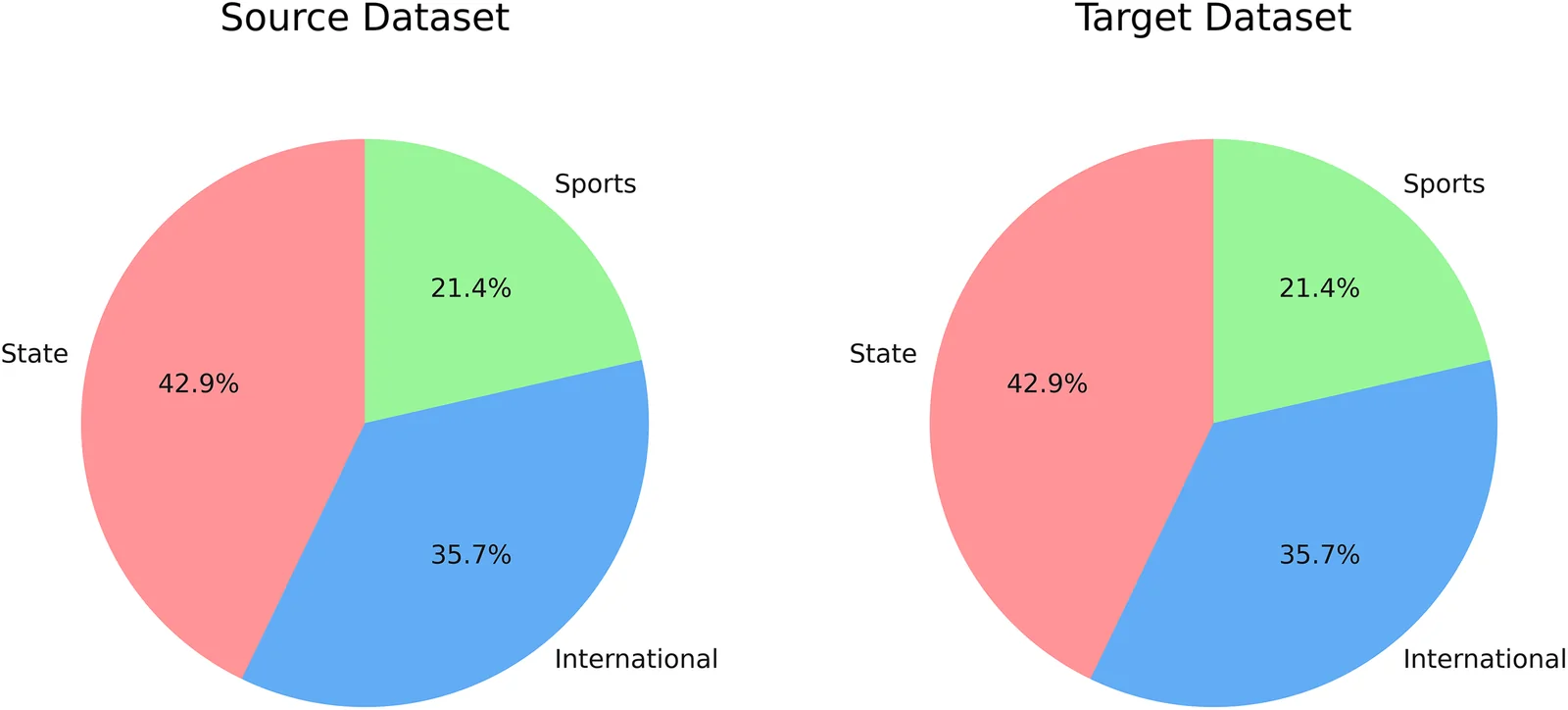
Distribution of data for domain adaptation for source and target domains of the custom dataset.

### Data preprocessing

Several preprocessing techniques were applied to clean and refine the custom Bengali news summarization dataset to ensure high-quality and structured input for training. A custom tokenizer specifically designed for Bengali text was used to convert text into tokenized sequences. This tokenizer was applied to the training and test datasets before feeding them into the transformer-based models. Tokenization ensured the model could efficiently process Bengali text and generate summaries effectively. Since the dataset was collected from various online sources, it contained redundant data entries that could impact training performance. To mitigate this, duplicate and null values were removed, ensuring only unique and complete data points were included.

Another important preprocessing step was resampling to address class imbalance in different categories. The dataset was divided into state, international, and sports categories, but the state category contained disproportionately more samples. To ensure fair representation across all categories, resampling techniques from the scikit-learn library were applied. Text formatting and normalization were performed to maintain consistency. All text was standardized to ensure uniform representation before being used for training. This included converting all input text to lowercase while preserving proper nouns. Bengali numerical values were converted to a standardized format to maintain consistency across different records. After applying these preprocessing techniques, the dataset was finalized with 21,512 structured text-summary pairs, ensuring clean and well-processed input for training the transformer-based summarization models.

### Transformer model architecture

In this work, two transformer-based architectures, mT5 [35] and Flan-T5 [36], have been used, which are particularly suited for sequence-to-sequence tasks-based text summarization. These models leverage an encoder-decoder structure, ensuring robust multilingual data handling and task-specific nuances. The process begins with the input sequence $X_{i}=\{x_{1},x_{2},...,x_{L}\}$, representing Bangla text that has been preprocessed and tokenized. Each tokenized sequence is converted into embeddings $T_{i}\in {\mathbb{R}}^{L\times d}$, where *L* is the fixed sequence length and *d* represents the embedding dimension. Positional encodings $P_{i}\in {\mathbb{R}}^{L\times d}$ are added to these embeddings, resulting in $E_{i}=T_{i}+P_{i}$, which captures both semantic and positional information.

The encoder processes $E_{i}$ through multiple layers of self-attention and feed-forward networks, generating latent representations $h_{i}=\{h_{1},h_{2},...,h_{L}\}$, where each $h_{i}\in {\mathbb{R}}^{m}$ encapsulates the semantic and contextual features of the input sequence. These latent vectors are passed to the decoder, which uses cross-attention mechanisms to focus on relevant parts of $h_{i}$ while generating the summary. The decoder predicts the output sequence ${\hat{Y}}_{i}=\{{\hat{y}}_{1},{\hat{y}}_{2},...,{\hat{y}}_{L^{\prime }}\}$, where $L^{\prime }$ is the length of the summary. Each decoder output is transformed into a probability distribution over the vocabulary using a softmax function, ensuring accurate token prediction at each step.

The mT5 model, trained on a diverse multilingual dataset, enables the framework to handle Bengali text effectively by providing language-agnostic and context-aware representations. Flan-T5, fine-tuned on task-specific datasets, enhances summarization performance by leveraging instruction-tuned knowledge. Together, these models form a robust foundation for the summarization pipeline, which is refined through domain adaptation mechanisms. Fine-tuning is performed using the XLSUM Bangla dataset and custom datasets specific to domains, e.g., state, international, and sports, ensuring adaptability to closed-set contexts. The output probabilities for each token are computed as:


$$\begin{array}{c} P({\hat{y}}_{t}|{\hat{y}}_{<t},h_{i})=\text{Softmax}(W_{o}z_{t}), \end{array}$$ (2)

where $z_{t}$ represents the decoder output at time step *t*, and $W_{o}$ is the output projec*t*ion matrix. To train the model, the cross-entropy loss for summary generation is defined as:


$$\begin{array}{c} L_{\text{CE}}=-\frac{1}{N}\sum_{i=1}^{N}\sum_{t=1}^{L^{\prime }}\log P({\hat{y}}_{t}|{\hat{y}}_{<t},h_{i}), \end{array}$$ (3)

where *N* represents the batch size. This loss ensures the generated summaries closely match the reference outputs. By combining these mechanisms with domain-specific fine-tuning, the proposed framework achieves robust and adaptable Bangla text summarization.

### Training LLM models on Bengali domain for text summarization

In this study, two large language models—mT5 and Flan-T5—were used for Bengali domain text summarization. For training, the Bengali split of the XLSUM dataset available on Hugging Face [37], containing 10.1k rows, was utilized. Initially, a tokenizer specifically optimized for the Bengali language was developed. The dataset was first converted into a text corpus, where the ‘text’ and ‘summary’ columns were extracted and saved in ‘.txt’ format to form a single combined corpus. All texts were concatenated into a single string for tokenizer training. The SentencePiece model was trained on the XLSUM Bengali corpus. SentencePiece, a subword tokenizer and detokenizer for neural text generation, enables direct training from raw text without pre-tokenization and supports segmentation algorithms such as Byte-Pair Encoding and unigram language modeling.

After training the SentencePiece model on the XLSUM Bengali corpus, its vocabulary tokens were merged with those of the T5 model to enhance Bengali vocabulary understanding within the T5-based tokenizer. The Flan-T5 tokenizer was loaded and reinitialized with the Bengali SentencePiece model, incorporating tokens such as <s>,  </s>, < pad > , and <unk > . The customized tokenizer was saved for domain-specific fine-tuning of the Flan-T5 model on Bengali text.

Following tokenization, the Flan-T5 model was trained on the training set of the Bengali split of the XLSUM dataset, while the test split was retained as unseen data for later evaluation. Fig 4 illustrates the text and summary length distributions for both the training and test sets. The Flan-T5 model and the custom tokenizer were loaded, and special tokens such as <pad> were added to the tokenizer. The model’s token embeddings were resized to align with the updated tokenizer. The XLSUM dataset was processed by tokenizing the ‘text’ and ‘summary’ columns using the custom tokenizer and applying the map function from the datasets library, completing the preprocessing phase.

**Fig 4:**
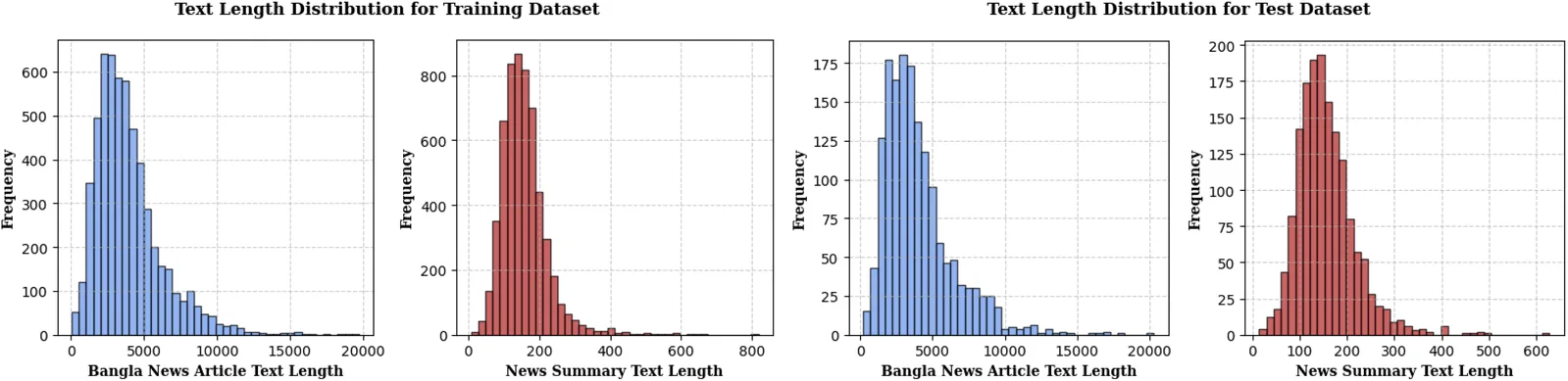
Text-length distributions in the XLSUM dataset for the training and test datasets.

The Flan-T5 model was trained for three epochs with a learning rate of 5e-5, achieving training and evaluation losses of 1.5500 and 1.4400, respectively. The generated summaries yielded a BLEU score of 0.001 and an ROUGE score of 0.16 compared to the reference summaries. The mT5 model was trained on the same dataset for ten epochs with a learning rate of 2e-5, resulting in a training loss of 3.5655 and a validation loss of 1.1406. Evaluation on the unseen test split of the XLSUM dataset produced ROUGE-1, ROUGE-2, and ROUGE-L scores of 0.21, 0.09, and 0.20, respectively, along with a BLEU score of 0.17 and a BERTScore of 0.72.

### Domain adaptation in closed-set context

To bridge the gap between the source and target domains, the suggested methodology uses domain adaptation in a closed-set context. In this case, both the source and target domains have the same label spaces, which encompass three distinct categories: state, international, and sports. By avoiding additional complexity due to label mismatch, this configuration guarantees that the model only concentrates on learning invariant features across domains. For domain adaptation, the applied LLM models, i.e., mT5 and Flan-T5, were first trained on the XLSUM Bengali corpus and on the Bengali split of the XLSUM dataset for text summarization. The BanglaMCT7 dataset was used as the source domain, and a custom dataset derived from web scraping Bengali newspaper articles was used as the target domain. Both datasets include three overlapping domains, including state, international, and sports, ensuring a uniform label space for closed-set domain adaptation.

These three categories were curated from the BanglaMCT7 dataset and merged to balance data distribution, reducing model bias. The final proportions of 42.9% for state, 35.7% for international, and 21.4% for sports were used for training the source data. Both mT5 and Flan-T5 were trained on these merged categories to provide general domain knowledge. Corresponding subsets from the custom dataset were extracted and used to fine-tune the source models individually for each domain (state, international, and sports). This two-stage process enabled the models to acquire more focused, domain-specific representations. The resulting three specialized models were evaluated on the test split using ROUGE, BLEU, and BERTScore metrics.

The first step in the closed-set domain adaptation process encoder consists of extracting semantic information from the input text and using it to generate latent representations $h_{i}$. The objective is to align the feature distributions of the source and target domains in this shared label space because the label spaces are identical, and the domain classification and domain confusion losses are used to accomplish this alignment. The domain discriminator calculates probabilities $P_{D}(z_{i}|h_{i})$ for a given domain label $z_{i}$, where $h_{i}$ represents the latent representation. The domain classification loss, depicted as:


$$\begin{array}{c} L_{D}=-\frac{1}{N}\sum_{i=1}^{N}[z_{i}\log({\hat{z}}_{i})+(1-z_{i})\log(1-{\hat{z}}_{i})], \end{array}$$ (4)

ensures that the discriminator accurately determines if a sample is associated with the target domain or the source domain.

Domain-specific features in the latent space $h_{i}$ must be minimized in the closed-set context. This is accomplished by using a gradient reversal layer (GRL) in the domain confusion loss, where the GRL encourages the encoder to generate domain-invariant features by negating the gradient signal from the domain discriminator. The domain confusion loss is expressed as:


$$\begin{array}{c} L_{DC}=-\frac{1}{N}\sum_{i=1}^{N}\log P_{D}(z_{i}|h_{i}), \end{array}$$ (5)

where the probability that $h_{i}$ belongs to a certain domain is quantified by $P_{D}(z_{i}|h_{i})$. The encoder aligns the feature distributions of the source and target domains within the shared label space by minimizing $L_{DC}$.

In order to accomplish the summarization process, the decoder optimizes the summaries using the cross-entropy loss $L_{CE}$ by processing the latent features $h_{i}$. The core objective of the model is to minimize the total loss:


$$\begin{array}{c} L_{\text{total}}=L_{CE}+\lambda_{1}L_{D}+\lambda_{2}L_{DC}, \end{array}$$ (6)

where the hyperparameters $\lambda_{1}$ and $\lambda_{2}$ regulate the domain adaptation losses’ contributions. This total loss maintains the semantic consistency necessary for summarization while ensuring that the model learns domain-invariant features.

The closed-set context streamlines the adaptation process by eliminating the necessity of mapping between different label spaces, enabling the model to concentrate on minimizing the divergence between the source and target feature distributions. In the zero-shot domain adaptation setting, the model’s generalization ability using the invariant characteristics learned during training is tested on the target domain without any additional fine-tuning, while in the fine-tuning domain adaptation setting, the model is trained on the target domain to improve its performance for subtleties unique to the target domain itself. This methodology guarantees efficient domain adaptation without compromising the caliber of generated summaries. The proposed technique’s robustness and adaptability are demonstrated by the use of metrics such as ROUGE, BLEU, and BERTScore by assessing summarization performance across domains.

In general, domain adaptation strengthens the quality of summarization by refining large language models on domain-specific data, which enables them to comprehend context, specialized vocabulary, and subtle structural discrepancies in a refined manner. Due to a lack of domain-relevant training data, general-purpose models frequently struggle with accuracy, producing imprecise or inaccurate summaries; adapting to a particular domain allows models to produce summaries with more cohesive structures, reduced hallucinations, and increased content relevancy. In low-resource languages, such as Bengali, domain adaptation is particularly significant since it substantially improves performance in both zero-shot and fine-tuning settings.

### Adapter-based fine-tuning for parameter-efficient domain adaptation

To overcome the computational overhead of full fine-tuning and improve training efficiency, we propose an adapter-based parameter-efficient fine-tuning (PEFT) approach built on top of the Flan-T5 source-domain model. In this setup, all base model parameters are frozen, and lightweight adapter layers are inserted into each transformer block. Each adapter consists of a down-projection layer that compresses the hidden representation into a smaller bottleneck dimension, followed by a non-linearity and an up-projection layer that restores it to its original size. These adapters are trained while the base model remains frozen, allowing efficient learning of domain-specific features without catastrophic forgetting.

We implemented this architecture using the Hugging Face peft library with LoRA (Low-Rank Adaptation) configuration, setting the rank r=16,α=32, and dropout = 0.1, targeting the attention query and value matrices with 3 epochs. The resulting model contains 77.6 million total parameters, with only 688,128 (0.89%) trainable parameters, achieving a 99% reduction in trainable weights compared to standard fine-tuning. This modular design allows adapters to be trained and stored per domain (e.g., sports, state, international), facilitating flexible reuse and combination across domains without retraining the entire model.

### Overall objective function

A comprehensive and holistic strategy that combines training dynamics of the mT5 architecture with domain adaptation-specific loss functions is employed in order to maximize the performance of the domain-adaptive Bengali text summarizing model. The main goal is directed by the **cross-entropy loss** (${ℒ}_{CE}$), which penalizes differences between the ground truth tokens $y_{t}^{*}$ and the predicted tokens $y_{t}$ to ensure efficient learning of the summary generation job. This has the following mathematical definition:


$$\begin{array}{c} {ℒ}_{CE}=-\frac{1}{T}\sum_{t=1}^{T}\log P(y_{t}^{*}|y_{<t},x), \end{array}$$ (7)

where the sequence length is indicated by *T* and the input text is represented by *x*. A **domain classification loss** (${ℒ}_{D}$) that uses domain labels z∈{0,1} is introduced to facilitate domain adaptation. This binary cross-entropy loss, which can be written as follows, motivates the model to differentiate between domains:


$$\begin{array}{c} {ℒ}_{D}=-\frac{1}{N}\sum_{i=1}^{N}\left[z_{i}\log{\hat{z}}_{i}+(1-z_{i})\log(1-{\hat{z}}_{i})\right], \end{array}$$ (8)

where *N* is the batch size, and ${\hat{z}}_{i}$ is the predicted domain label. In order to promote domain-invariant latent representations, we employ a **domain confusion loss** (${ℒ}_{DC}$), which is implemented using a gradient reversal layer. This is described as:


$$\begin{array}{c} {ℒ}_{DC}=-\frac{1}{N}\sum_{i=1}^{N}\log P_{D}(z_{i}|h_{i}), \end{array}$$ (9)

where the hidden representation of the *i*-th input is denoted by $h_{i}$. These elements are used with weighting factors $\lambda_{1}$ and $\lambda_{2}$ in the final **total loss** to balance the contributions of domain adaptation, as indicated by:


$$\begin{array}{c} {ℒ}_{total}={ℒ}_{CE}+\lambda_{1}{ℒ}_{D}+\lambda_{2}{ℒ}_{DC}. \end{array}$$ (10)

To guarantee uniform sequence lengths *L*, the input sequence *X* and target summary *Y* are tokenized using a tokenizer *T* during the mT5 model’s training procedure, as follows:


$$\begin{array}{c} T(X)=\text{Pad}(\text{Truncate}(X,L),L). \end{array}$$ (11)

The AdamW optimizer with weight decay λ is used to optimize the model, while cosine annealing with restarts is applied to schedule the learning rate:


$$\begin{array}{c} \eta_{t}=\eta_{\text{min}}+\frac{1}{2}(\eta_{\text{max}}-\eta_{\text{min}})\left(1+\cos\left(\frac{t\pi }{T_{\text{cur}}}\right)\right), \end{array}$$ (12)

where the current cycle length has been designated by *T*_cur_. To replicate a larger batch size, gradient accumulation is adopted:


$$\begin{array}{c} \text{Effective Batch Size}=\text{Per Device Batch Size}\times G, \end{array}$$ (13)

where the accumulation steps are symbolized by *G*. These techniques ensure strong domain adaptation and superior summarization performance when coupled with domain-specific loss formulations.

### Development of user interface for the summary generation

To create a user-friendly web interface, illustrated in Fig 5, for the proposed Bengali text summarization system, where users can input long Bengali text and receive a concise summary, web development frameworks, Flask, were used for backend development. Front-end technologies such as HTML, CSS, and JavaScript were employed to design the interface. The backend handles requests from the front-end by loading the LLM models and performing text summarization. The backend receives the input text via the POST request method, the custom tokenizer from Hugging Face tokenizes the input text into token IDs, and the model generates a summary based on the input. The backend sends output to the front-end as JSON. Consequently, HTML defines the structure of the page, input box, output text area, and button for triggering summarization and CSS handles the styling of the input box, output area, and button for a visually appealing interface. The fetch API of JavaScript sends requests to the backend and Flask processes the request, runs the model and returns the result in JSON format. JavaScript parses the JSON response and updates the DOM to display the output summary in the UI, aiding users quickly and simply creating insightful summaries from long, complex Bengali texts.

**Fig 5:**
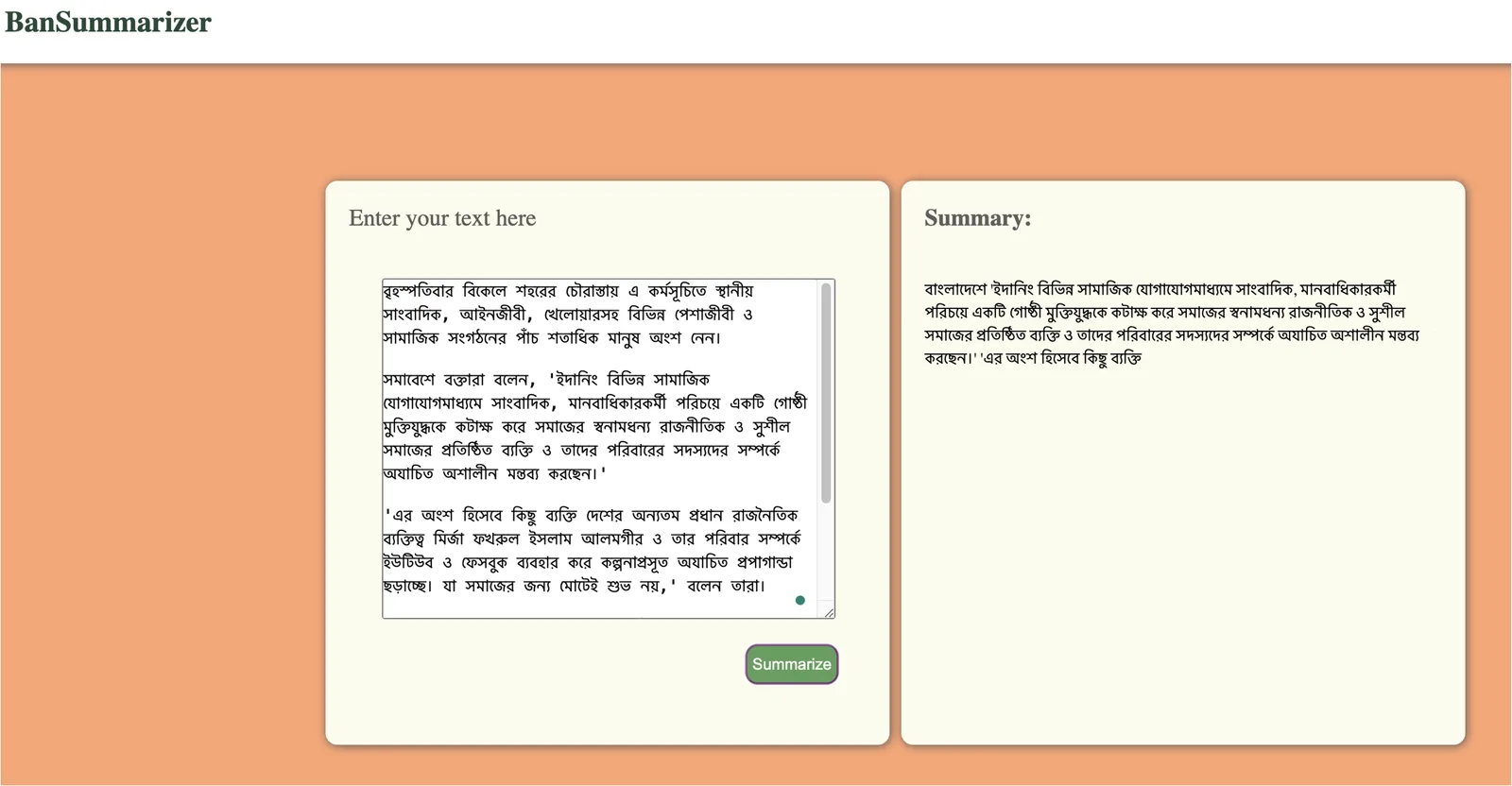
Web interface of the proposed Bengali text summarization system.

## Results and discussion

This section discusses the results of the proposed Bengali text summarization leveraging large language models and domain adaptation on a closed-set study. ROGUE, BLEU and BERT scores were used as evaluation parameters to assess the performance of the different LLM models for both Bengali text summarization and domain adaptation on fine-tuning and zero-shot settings.

### Training metrics and summary for Bengali text summarization

Table 4 presents a comprehensive training summary including hyperparameters and training outcomes of the applied LLM models to summarize the Bengali texts in the dataset. The training process involved fine-tuning these models on the Bengali corpus to evaluate their performance in generating summaries. The hyperparameter settings used for training the Flan-T5 and mT5 models are presented in Table 5. Flan-T5 was trained with a learning rate of $5\times 10^{-5}$ and a batch size of 8. The mT5 model was trained for 10 epochs with a learning rate of $2\times 10^{-5}$, batch size of 2, and 4 gradient accumulation steps. All the hyperparameters have been fixed employing the Optuna tool. For Domain Adaptation, we used 80% for training and 20% for testing.

**Table 4:** Training summary of the applied models.

Model	Learning Rate	Epochs	Train Loss	Val Loss	#Params	Train Time	Inference Time
Flan-T5	5e-5	3	1.5500	1.4400	~80M	23 minutes	17 ms
mT5	2e-5	10	3.5655	1.1406	~300M	88 minutes	18.9 ms

**Table 5:** Hyperparameter settings used for Flan-T5 and mT5 models.

Hyperparameter	Flan-T5	mT5
Model variant	Flan-T5-small	mT5
Optimizer	AdamW	AdamW
Learning rate	$5\times 10^{-5}$	$2\times 10^{-5}$
Training batch size	8	2
Evaluation batch size	8	2
Gradient accumulation steps	–	4
Weight decay	0.01	0.01
Evaluation strategy	Epoch-wise	Epoch-wise
Checkpoint saving	Each epoch	Every 5000 steps
Maximum checkpoints retained	3	1
Learning rate scheduler	Default	Cosine with restarts
Warmup steps	–	1000
Logging steps	100	200
Mixed precision	Disabled	Disabled
Sequence generation for evaluation	Enabled	Enabled

The Flan-T5 and mT5 LLM models were initially trained on the Bengali split of the XLSUM dataset. The mT5 model was trained on an epoch of 10 with a learning rate of 2e-5. The model being trained for 10 epochs ensured sufficient exposure to the training data for convergence without overfitting, while the choice of the learning rate as 2e-5 was optimal for minimizing overfitting and achieving convergence within the 10 epochs. The training loss and validation loss were reported to be 3.56 and 1.14, respectively. This training loss is generally acceptable for complex tasks, such as text summarization, while the validation loss being slightly lower than the training loss indicates effective generalization, suggesting that the model performs well on unseen data. The Flan-T5 model was trained on an epoch of 3 with a learning rate of 5e-5. This relatively high learning rate suits the smaller number of epochs, providing faster updates to the model parameters. The gap between the training loss and validation loss indicates a well-regularized model. The training metrics for the mT5 and Flan-T5 models are shown in Figs 6 and 7, respectively. Despite both LLM models showing strong generalization capabilities, the mT5 model’s lower validation loss suggests it is more robust in adapting to the complexities of a low-resource language, Bengali.

**Fig 6:**
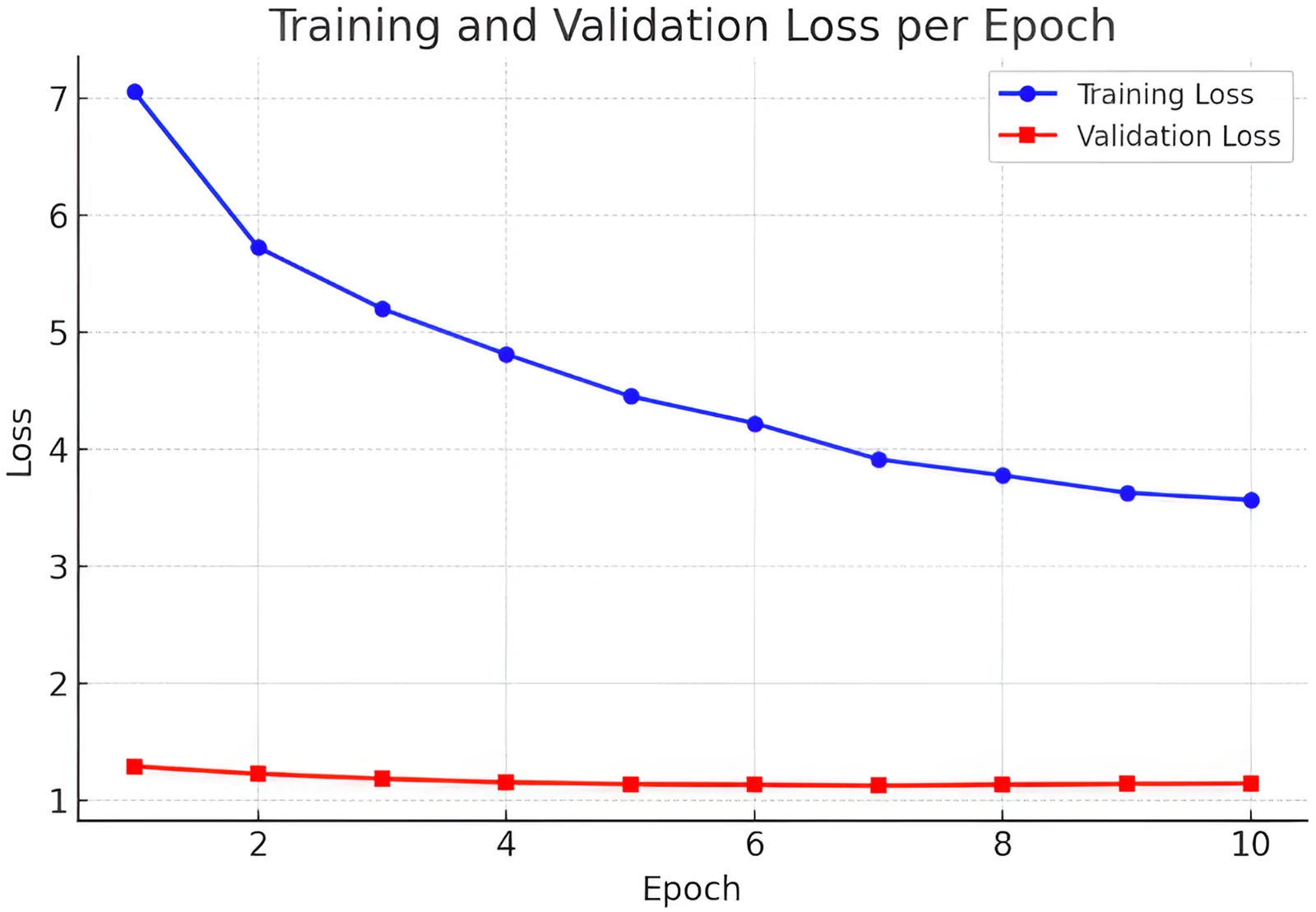
Training metrics for mT5 model.

**Fig 7:**
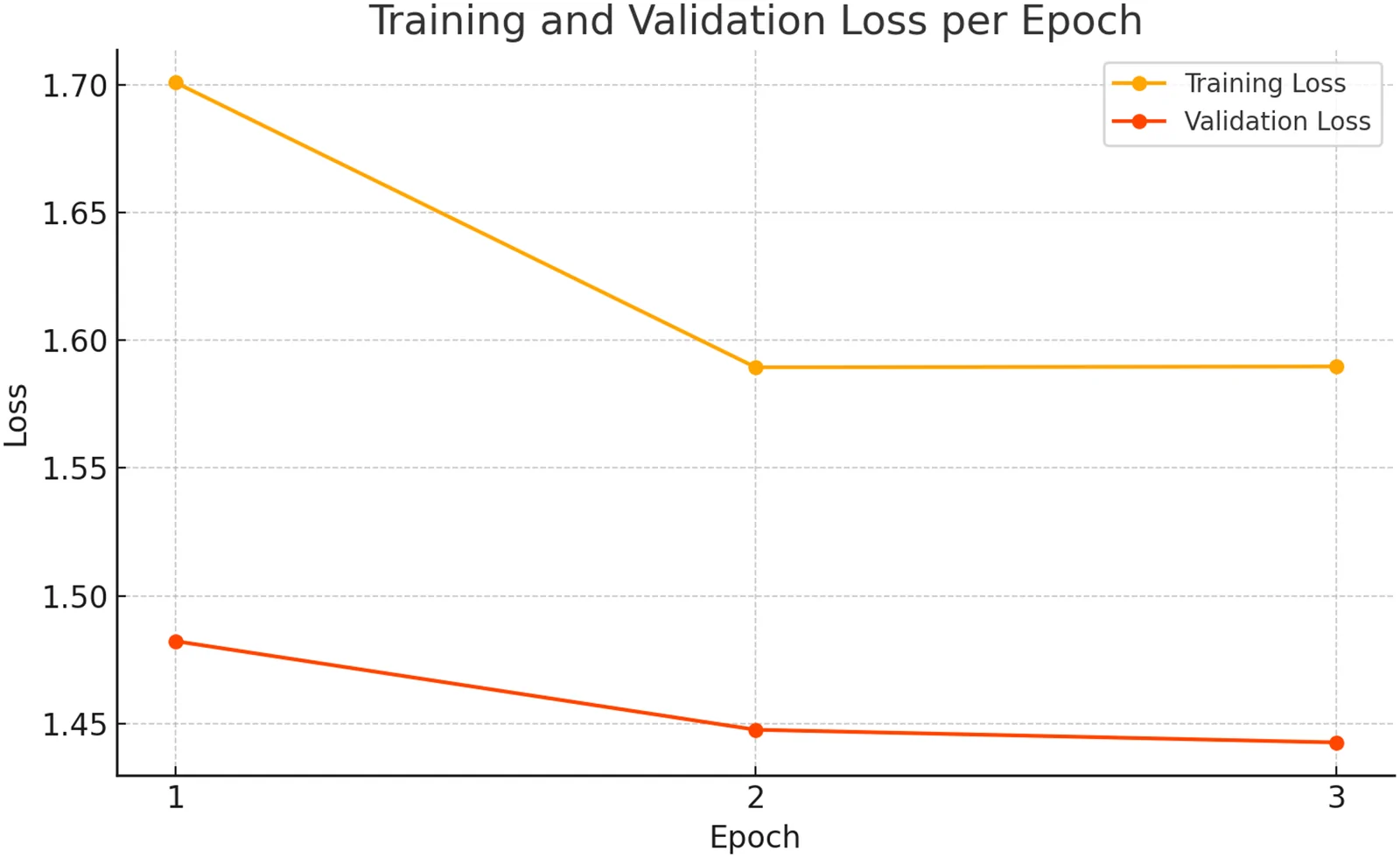
Training metrics for Flan-T5 model.

The evaluation parameters of the two LLM models after training were examined as demonstrated in Table 6, providing important context about the diagnostic capacities of the models. The models were assessed using three evaluation metrics: ROUGE, BLEU, and BERTScore.

**Table 6:** Evaluation parameters of the models for Bengali text summarization.

Models	ROUGE-1	ROUGE-2	ROUGE-L	BLEU	BERT
Flan-T5	0.15	0.04	0.10	0.17	0.76
mT5	0.24	0.09	0.20	0.17	0.72

Following the training process, the Flan-T5 and mT5 models’ performance was evaluated on the XLSUM dataset’s test split, which served as unseen data during training. The weighted ROUGE F1-measure scores were employed as the primary metric for comparing model performance. Among the two LLM models, the mT5 model achieved the highest ROUGE scores on the XLSum dataset, with a ROUGE-1 score of 0.21, ROUGE-2 score of 0.09, and ROUGE-L score of 0.20. The mT5 model outperformed the Flan-T5 model across all evaluation metrics, attaining a BLEU score of 0.17 and a BERTScore of 0.72. Flan-T5, while performing comparatively lower, achieved a BERTScore of 0.60, suggesting a moderate level of semantic similarity with the reference summaries. These evaluation parameter results indicate that mT5, with its multilingual training base, is better suited for Bengali text summarization tasks in this setting. Table 7 presents the summaries generated by both LLM models alongside the reference summary for a sample text.

**Table 7:** Sample of generated summaries using the two models.

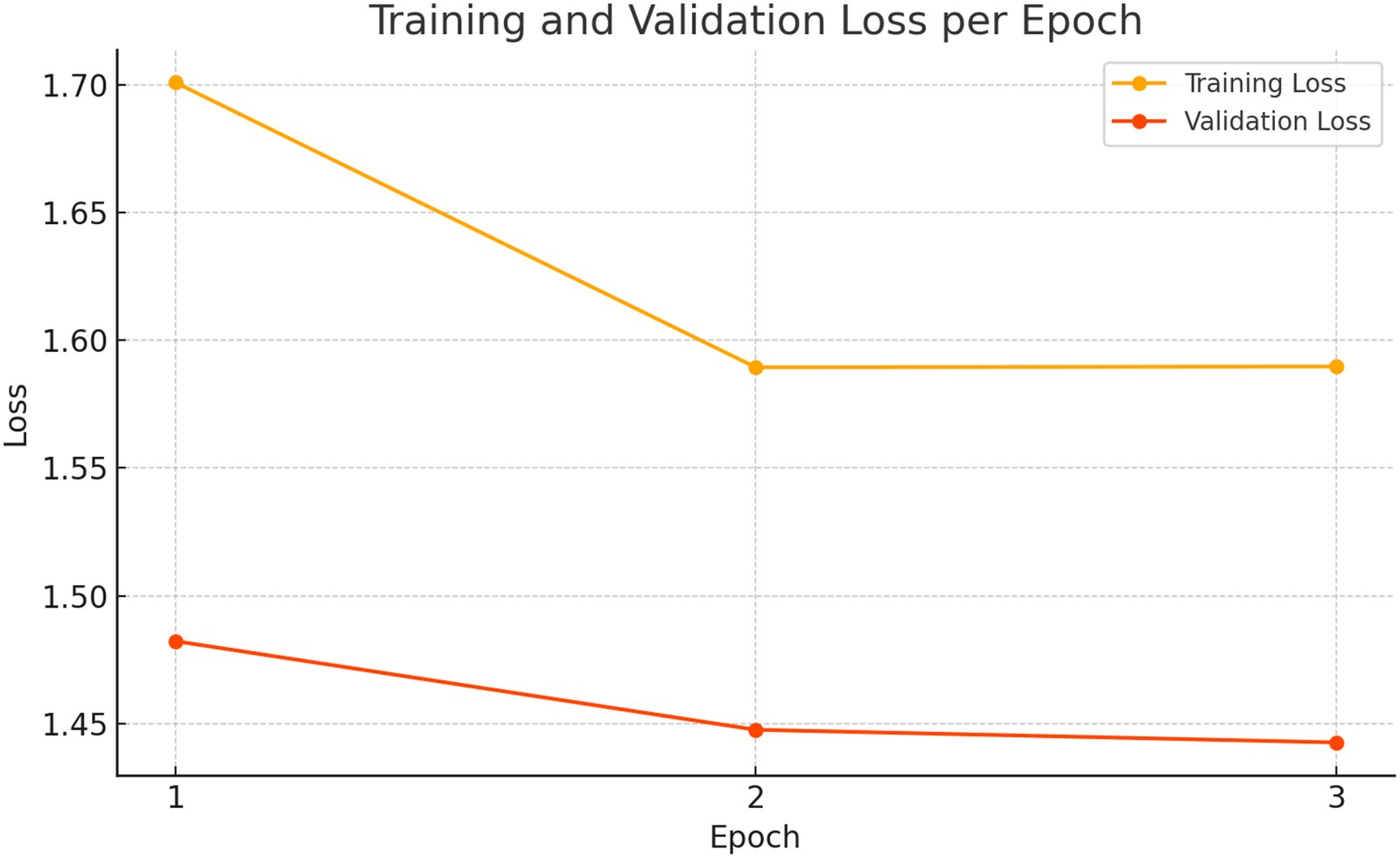

In addition to the multilingual LLMs, Bengali-specific models, **Bangla-BERT** and **BanglaT5**, were incorporated for comparative analysis. In contrast to the sequence-to-sequence models Flan-T5 and mT5, Bangla-BERT is a transformer-based encoder model that has been pretrained exclusively on an extensive Bengali corpus. BanglaT5 is a sequence-to-sequence model trained solely on Bengali text, designed to generate fluent and contextually rich summaries by leveraging native linguistic patterns. In order to better capture grammatical and semantic characteristics unique to the Bengali language, native Bengali models are evaluated alongside multilingual LLMs. Table 8 illustrates the evaluation parameters for the BanglaBERT and BanglaT5 models.

**Table 8:** Evaluation parameters of BanglaBERT and BanglaT5 models for comparative analysis in Bengali text summarization.

Models	ROUGE-1	ROUGE-2	ROUGE-L	BLEU	BERT
BanglaBERT	0.08	0.02	0.06	0.03	0.94
BanglaT5	0.09	0.02	0.08	0.008	0.64

The evaluation results for Flan-T5, mT5, and Bangla-BERT reveal distinct strengths across metrics. While the mT5 model obtained the greatest ROUGE and BLEU scores, demonstrating superior lexical and structural alignment and Flan-T5 demonstrated competitive performance with balanced outcomes on all metrics, Bangla-BERT showed a deeper semantic comprehension of Bengali material because of its significantly higher BERTScore (0.94). In comparison, BanglaT5 presented comparatively lower results on all parameters, indicating a limited capacity for generalization. These findings demonstrate how multilingual and monolingual models complement each other in Bengali text summarization, with Bangla-BERT capturing more semantic nuances and mT5 and Flan-T5 excelling at surface-level similarities.

### Evaluation parameters for domain adaptation in different settings

The LLM models were assessed using five evaluation metrics for domain adaptation: ROUGE, BLEU, BERTScore, Distinct-1, Distinct-2 and Self-BLEU.

ROUGE is a set of metrics used to evaluate the quality of summaries or translations by comparing them to reference summaries or translations. It measures the overlap of n-grams (contiguous sequences of n items, typically words) between the generated summary and the reference summaries. The type of ROUGE metrics used for this research is as follows.

- ROUGE-N: This measures the overlap of n-grams between the generated summary and reference summaries. It includes ROUGE-1 (unigrams), ROUGE-2 (bigrams), ROUGE-3 (trigrams), etc.
  $$\begin{array}{c} ROUGE\text{-}N=\frac{\sum_{gram_{n}\in Ref}\min({\text{Count}}_{gen}(gram_{n}),{\text{Count}}_{ref}(gram_{n}))}{\sum_{gram_{n}\in Ref}{\text{Count}}_{ref}(gram_{n})} \end{array}$$ (14)
- ROUGE-L: This measures the longest common subsequence (LCS) between the generated summary and reference summaries.

$$\begin{array}{c} ROUGE\text{-}L=\frac{LCS(Gen,Ref)}{\left|Ref\right|} \end{array}$$ (15)

BLEU is a metric for evaluating the quality of machine-translated text by comparing it to one or more reference translations. It measures the precision of n-grams (contiguous sequences of n items, typically words) in the generated translation compared to the reference translations. BLEU computes a precision score for each n-gram up to a certain length (usually up to 4 grams) and combines them using a weighted geometric mean.


$$\begin{array}{c} BLEU=BP\cdot \exp(\sum_{n=1}^{N}w_{n}\log p_{n}), \end{array}$$ (16)

where $p_{n}$ is the precision of n-grams, $w_{n}$ are weights, and *BP* is the brevity penalty.

BERTScore uses BERT embeddings to evaluate the similarity between the generated summary and the reference summary at a semantic level, rather than just surface-level word overlap. It calculates precision, recall, and F1-score by computing cosine similarities between BERT embeddings of words in the generated and reference summaries, and matches words based on these similarities, providing a deeper semantic comparison.


$$\begin{array}{c} BERTScore=F_{1}\text{-score based on cosine similarity of token embeddings} \end{array}$$ (17)

Distinct-1/2 is a measure set employed to assess the diversity of created text by quantifying the ratio of unique n-grams in the produced summary. Distinct-1 and Distinct-2 concentrate on the originality and diversity of words or word sequences in the generated summaries, as opposed to ROUGE or BLEU, which gauge resemblance to a reference.

- Distinct-1: Calculates the proportion of distinct unigrams (individual words) to the resulting summary’s word count.
- Distinct-2: Calculates the proportion of distinct bigrams—word pairs that follow one another—to all bigrams in the summary.

$$\begin{array}{c} Distinct\text{-}n=\frac{\text{Number of unique n-grams in the generated summary}}{\text{Total number of n-grams in the generated summary}} \end{array}$$ (18)

Self-Bleu is a metric used to assess how repetitive the summaries that are created inside a corpus are. Each generated summary’s BLEU score is calculated using all other generated summaries as references. Self-BLEU measures redundancy across outputs rather than within a single summary, making it a useful addition to distinct metrics.


$$\begin{array}{c} Self\text{-}BLEU(Gen_{i})=BLEU(Gen_{i},\{Gen_{1},...,Gen_{i-1},Gen_{i+1},...,Gen_{n}\}) \end{array}$$ (19)

For domain adaptation, the mT5 and Flan-T5 LLM models previously trained on the XLSUM Bangla corpus and fine-tuned on the XLSUM (Bengali) dataset for text summarization were employed. The BanglaMCT7 dataset was utilized as the source dataset, while a custom dataset served as the target dataset. Under the closed-set domain adaptation approach, three domains, e.g., state, international, and sports, were selected from both the BanglaMCT7 and custom datasets. These domain-specific subsets were merged separately to constitute the source and target domains, respectively. The models adapted to these three specific domains were evaluated under both fine-tuning and zero-shot settings. Table 9 demonstrates a detailed overview of the evaluation metrics (ROUGE, BLEU, and BERTScore) obtained in these experimental configurations for the two models.

**Table 9:** Evaluation parameters for different domains leveraging LLM models in different settings.

Models	State			International			Sports		
	ROUGE	BLEU	BERT	ROUGE	BLEU	BERT	ROUGE	BLEU	BERT
*Fine-Tuning Setting*									
**Flan-T5**	0.79	0.13	0.86	0.78	0.12	0.85	0.79	0.11	0.86
**mT5**	0.41	0.31	0.81	0.44	0.36	0.83	0.35	0.27	0.81
*Zero-Shot Setting*									
**Flan-T5**	0.57	0.38	0.85	0.67	0.47	0.89	0.59	0.34	0.88
**mT5**	0.65	0.47	0.84	0.45	0.38	0.84	0.33	0.23	0.82

In the fine-tuning setting, the models were initially trained on the source domain, which comprised the merged dataset of the three specific domains, i.e., state, international, and sports, from the BanglaMCT7 dataset. The target domain similarly consisted of the merged dataset of the same three domains from the custom dataset. The two base LLM models were first trained on the source domain and fine-tuned on the target domain to obtain ROUGE, BLEU, and BERTScore metrics separately for each domain. The evaluation results indicate that the Flan-T5 model outperformed the mT5 model regarding ROUGE and BERTScore, whereas the mT5 model achieved higher BLEU scores. Among the domains, the state domain demonstrated superior performance in the fine-tuning setting. Specifically, for the Flan-T5 model, the ROUGE score was 0.79, the BLEU score was 0.13, and the BERTScore was 0.86.

The zero-shot setting was applied to the models adapted to the three specific domains. In this configuration, the domain-specific models were evaluated on previously unseen, domain-specific datasets comprising 500 entries each for the state, international, and sports categories. These datasets were individually compiled by scraping content from online Bengali news portals. As required by the zero-shot setting, only inference was performed, and the models were evaluated using ROUGE, BLEU, and BERTScore metrics across the three domains. The evaluation revealed that the mT5 model performed best in the state domain, whereas the Flan-T5 model yielded superior results in the international and sports domains. The highest values were obtained for the international domain, where for the Flan-T5 model, the ROUGE, BLEU and BERT scores are 0.67, 0.47 and 0.89, respectively.

The models were evaluated using additional metrics, including distinct-1, distinct-2, and self-BLEU scores, to assess various aspects of the model’s performance beyond traditional accuracy-based evaluations. Table 10 presents the scores across three domains in both zero-shot and fine-tuning settings. The mT5 model outperforms Flan-T5 across almost all domains in terms of distinct and self-BLEU scores.

**Table 10:** Evaluation of generated summaries using Distinct-1/2 and Self-BLEU scores.

Models	State			International			Sports		
	Distinct-1	Distinct-2	Self-BLEU	Distinct-1	Distinct-2	Self-BLEU	Distinct-1	Distinct-2	Self-BLEU
*Fine-Tuning Setting*									
**Flan-T5**	0.30	0.82	0.14	0.34	0.83	0.11	0.28	0.68	0.23
**mT5**	0.69	0.95	0.17	0.64	0.93	0.21	0.60	0.94	0.22
*Zero-Shot Setting*									
**Flan-T5**	0.27	0.71	0.22	0.26	0.72	0.19	0.19	0.58	0.33
**mT5**	0.63	0.93	0.21	0.66	0.94	0.20	0.53	0.85	0.34

### External evaluation

In addition to the automatic evaluation metrics (ROUGE, BLEU, and BERTScore), an external evaluation study was conducted to assess the qualitative performance of the generated summaries. A Google Form was designed to collect participant judgments from 28 participants on summaries produced by the Flan-T5 and mT5 models across three domains, including State, International, and Sports. Their native language proficiency and familiarity with Bangla articles were taken into account. Each participant was presented with the original article, the reference summary, and the model-generated summary. They were asked to evaluate the generated summary on three key dimensions: Fluency — grammatical correctness and readability of the text, Adequacy / Informativeness — how well the summary captures essential content from the original text, and Factual Consistency — accuracy of facts relative to the source article. Participants rated each dimension on a five-point Likert scale (1 = Poor, 5 = Excellent). The collected responses were averaged to provide a holistic evaluation score for each model-domain combination, complementing the quantitative metric results.

Table 11 illustrates the average evaluation scores in fluency, adequacy, and factual consistency (1–5 scale) of 28 people for the Flan-T5 and mT5 models in 3 domains. Across all domains, both models achieved high fluency scores, indicating that the generated summaries were grammatically correct and naturally phrased in Bangla. The Flan-T5 model achieved the highest average score in the International domain (4.23), indicating strong performance in both readability and factual alignment. Meanwhile, the mT5 model slightly outperformed Flan-T5 in the State domain (4.17 vs. 4.14), suggesting better content adequacy in domain-specific summaries. In the Sports domain, both models showed comparable performance with average scores around 4.2, highlighting their robustness across different content types.

**Table 11:** External evaluation of generated summaries using fluency, adequacy, consistency, and average scoress.

Models	Domain	Fluency	Adequacy	Consistency	Avg. Score
**Flan-T5**	**State**	4.36	3.96	4.11	4.14
**mT5**	**State**	4.21	4.21	4.11	4.17
**Flan-T5**	**Sports**	4.29	4.18	4.18	4.21
**mT5**	**Sports**	4.43	3.93	4.14	4.16
**Flan-T5**	**International**	4.25	4.25	4.21	4.23
**mT5**	**International**	4.36	3.93	4.21	4.16

### Comparison of full fine-tuning vs. adapter-based fine-tuning

From the Table 12 it is noticeable that Adapter-based fine-tuning achieves similar performance with far fewer parameters on metrics- ROUGE and BLEU scores across 3 domains-sports, state and international. While the absolute performance is slightly lower, the adapter-based approach offers a trade-off between computational efficiency and generalization. This architecture enables efficient multi-domain transfer without retraining the entire model, representing a scalable and modular direction for Bengali domain-adaptive summarization.

**Table 12:** Comparison of full fine-tuning vs. adapter-based fine-tuning on Flan-T5 using ROUGE, BLEU.

Model	Domains	Trainable Params	ROUGE-1	ROUGE-2	ROUGE-L	ROUGE-L
Flan-T5 Full FT	3	77M	0.21	0.08	0.20	0.05
Flan-T5 + Adapter	3	0.69M	0.19	0.08	0.19	0.05

Unlike conventional full fine-tuning, where all model parameters are updated, adapter tuning introduces lightweight bottleneck modules within each transformer layer. This technique drastically reduces trainable parameters (<1% of total) while maintaining or improving domain adaptation performance. The adapter-augmented Flan-T5 model contained 77.6M total parameters, with only 0.89% (688K) trainable. Despite this reduction, the model achieved comparable ROUGE and BLEU scores to full fine-tuning while significantly improving training efficiency.

### Ablation study

An ablation study was conducted to analyze the contributions of various components within the Bengali text summarization models by eliminating or modifying specific elements of the methodology. Table 13 presents the results of this study, where experiments were performed under different conditions to evaluate their individual impacts. The first experiment examined the effect of pre-training on the performance of the Flan-T5 and mT5 models. The results indicated that models without pre-training exhibited significantly lower performance. In contrast, pre-trained models achieved substantially better evaluation metrics, particularly in ROUGE and BLEU scores, which were nearly twice as high as those of the non-pretrained models. The second experiment assessed the impact of fine-tuning on both models. Fine-tuning on domain-specific data, especially within the sports domain, improved performance compared to fine-tuning on generic data. The Flan-T5 model, fine-tuned on domain-specific data, achieved the best performance across all configurations, particularly concerning ROUGE and BLEU scores, highlighting its ability to generate more accurate and contextually relevant summaries. Although fine-tuning on generic data enhanced performance, it remained less effective than domain-specific fine-tuning for both models.

**Table 13:** Ablation study: Impact of pretraining and fine-tuning on performance metrics.

Experiment Type	ROUGE-1	ROUGE-2	ROUGE-L	BLEU	BERTScore
No Pretraining, trained from scratch (Flan-T5)	0.00	0.00	0.00	0.00	0.01
No Pretraining, trained from scratch (mT5)	0.13	0.03	0.11	0.12	0.74
Pre-trained model (Flan-T5)	0.38	0.30	0.37	0.20	0.80
Pre-trained model (mT5)	0.24	0.09	0.20	0.17	0.72
Fine-Tuning on domain-specific data – sports (Flan-T5)	0.54	0.45	0.54	0.32	0.85
Fine-Tuning on domain-specific data – sports (mT5)	0.36	0.23	0.32	0.27	0.81
Fine-Tuning on generic data (Flan-T5)	0.21	0.08	0.20	0.05	0.73
Fine-Tuning on generic data (mT5)	0.16	0.13	0.15	0.10	0.72

### Error analysis of model-generated summaries

A few errors were encountered during the training process, which are analyzed in this paragraph. The first issue noticed after the Flan-T5 and mT5 models generated summaries was the omission of key information. This issue was identified when some of the generated summaries did not contain important details from the original text, and proved challenging to decipher the meaning. This error was more frequently observed in the Flan-T5 model than in the mT5 model. One notable difference between the outputs of the two models was that the summaries produced by Flan-T5 were generally more concise than those generated by mT5. For instance, critical contextual elements such as team names and locations in the sports domain were occasionally omitted. For the economy category, important information, such as the GDP growth rate or inflation percentage, was sometimes omitted, which made the generated summary less meaningful. Other than that, some of the generated summaries had poor sentence fluency, as a few of them lacked natural flow and coherence. These were the few errors analyzed while performing the research.

### Robustness and statistical significance analysis

Table 14 presents the performance comparison between the baseline Flan-T5 model and the domain-specific models across three domains. The domain-adapted models consistently outperform the baseline across all domains. In the state domain, the proposed model improves ROUGE-1 from 0.331 to 0.563. In the international domain, the ROUGE-1 score increases from 0.313 to 0.685, and in the sports domain from 0.128 to 0.588.

**Table 14:** Performance comparison between baseline Flan-T5 and domain-adapted models across different domains.

Domain	Model	ROUGE-1	ROUGE-2	ROUGE-L
State	Base (Flan-T5)	0.331	0.254	0.325
	Domain Model	0.563	0.478	0.563
International	Base (Flan-T5)	0.313	0.247	0.311
	Domain Model	0.685	0.597	0.685
Sports	Base (Flan-T5)	0.128	0.070	0.122
	Domain Model	0.588	0.463	0.588

To validate the significance of the improvements, a paired t-test was conducted on the ROUGE-1 scores. As shown in Table 15, the improvements are statistically significant across all three domains, with all p-values below 0.001.

**Table 15:** Statistical significance analysis using paired t-test on ROUGE-1 scores.

Domain	t-statistic	p-value	Significance
State	5.84	< 0.001	Significant
International	10.65	< 0.001	Significant
Sports	22.67	< 0.001	Significant

### Explainability and interpretability analysis

Three explainability methods were employed to interpret the output of the domain-adaptive Flan-T5 Bengali text summarization model fine-tuned on Bangla sports news: LIME, SHAP, and BertViz. LIME provided token-level explanations with a high confidence score of 1.00, highlighting tokens such as মধ্যপ্রাচ্যের’, ‘দেশ’, ‘ওমানের’, ‘জাতীয়’, ‘লীগের’ which are geographical, national, and contextually meaningful, indicating a strong positive influence on the model’s output. Fig 8 presents the LIME output for the sample predictions. SHAP explained the model’s decision-making by quantifying the positive and negative contributions of each token. As shown in Fig 9, tokens such as ‘মধ্যপ্রাচ্যের’, ‘দেশ’, ‘জাতীয়’, ‘ওমানের’ demonstrated the most significant positive impact, while tokens such as ‘ডি,’ ‘টি,’ ‘করেছে,’ ‘যোগ্যতা’ negatively influenced the model’s predicted outcome. The base value of −19.0802 represents the model’s initial expected output prior to processing the input, while the final prediction score of 0.978326, close to 1, indicates strong model confidence and effective contextual comprehension. BertViz was used to visualize attention weights within the transformer architecture. Fig 10 illustrates the attention patterns for a sample input, where the model consistently assigned the highest attention to tokens such as ‘দেশ’, ‘বলাদেশ’, ‘জাতীয়’ across multiple attention heads. These findings are consistent with the insights obtained from both LIME and SHAP. While the employed XAI frameworks effectively reveal token-level importance and attention patterns, they are limited in capturing higher-level discourse or syntactic structures in Bengali text. English translations of the Bengali tokens displayed in the illustrated attention-head visualization are provided in Supplementary File S1.

**Fig 8:**
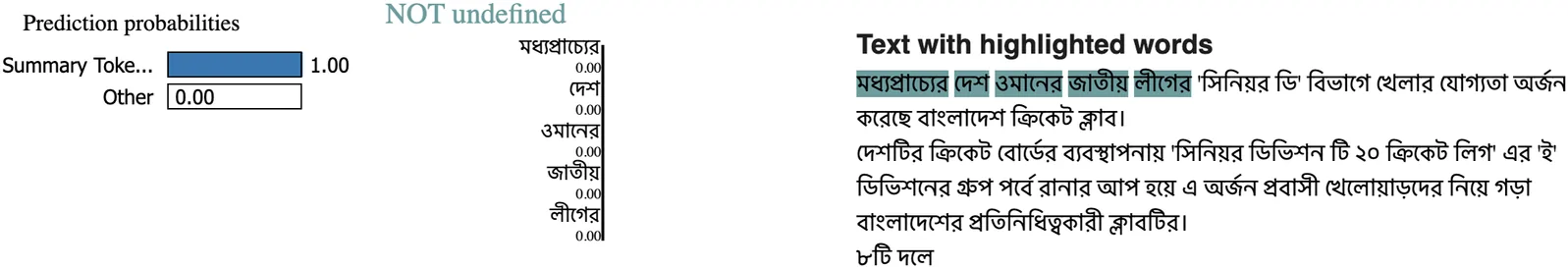
LIME visualization of token importance for Flan-T5 summary prediction in the sports domain.

**Fig 9:**
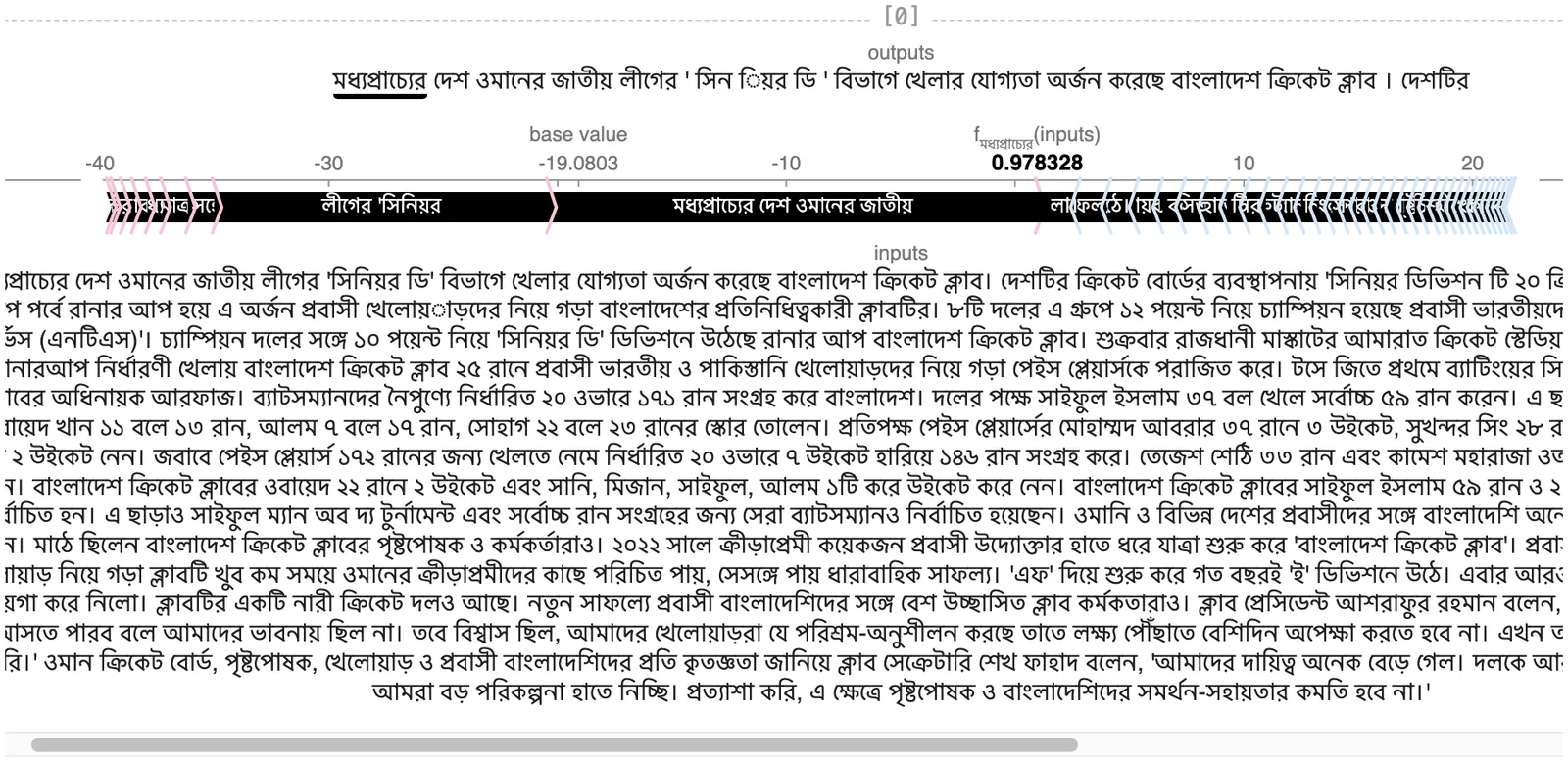
SHAP explanation of token-level contributions in Flan-T5 summary prediction for the sports domain.

**Fig 10:**
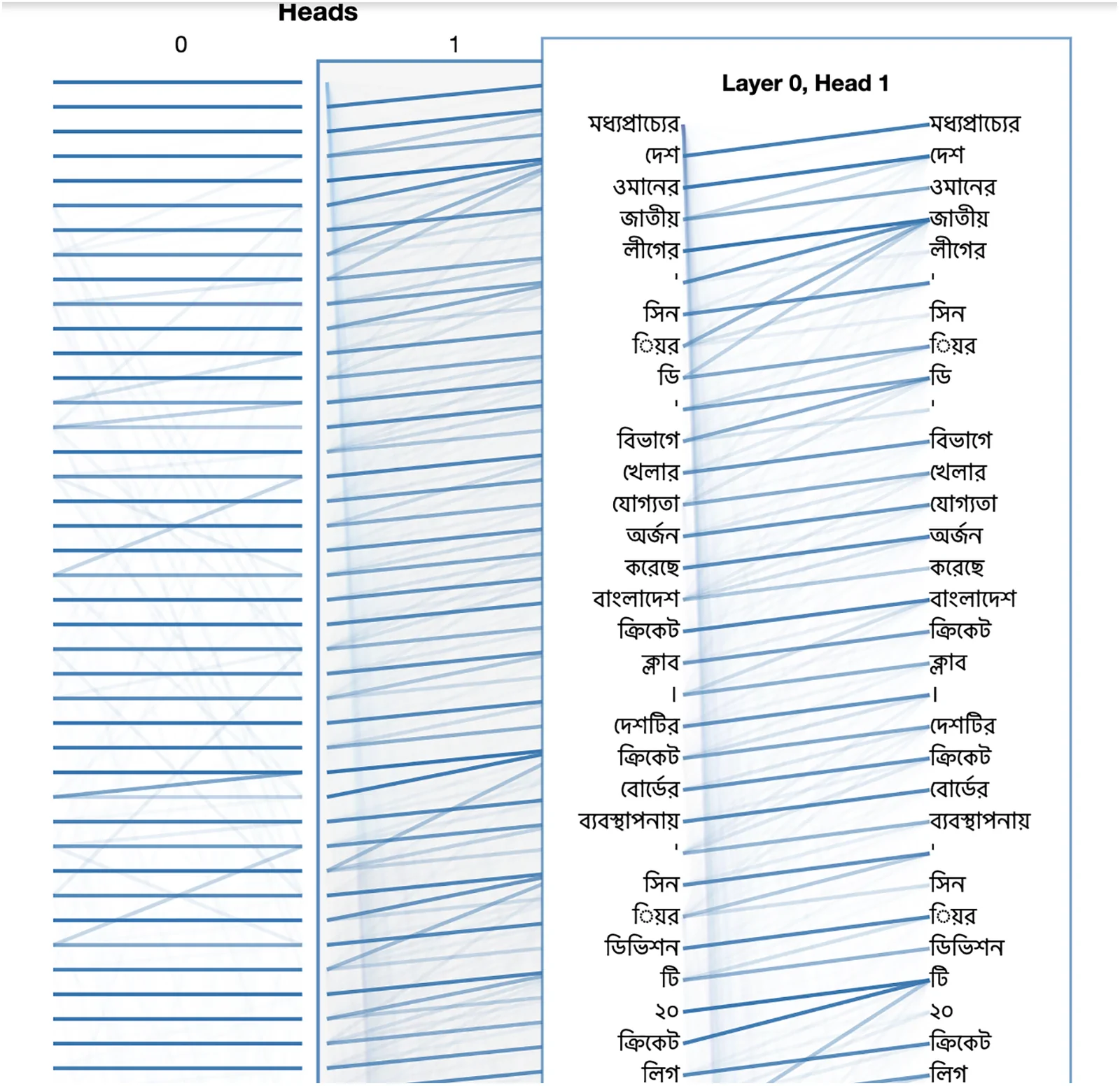
BertViz explanation of token-level contributions in Flan-T5 summary prediction for the sports domain.

## Discussion

This study aimed to evaluate the performance of two large language models, Flan-T5 and mT5, across multiple configurations, evaluation metrics, and domain-specific scenarios. Three primary evaluation metrics, including ROUGE, BLEU, and BERTScore, were employed to assess model performance in both fine-tuning and zero-shot settings. The growing need for effective natural language processing (NLP) tools for low-resource languages such as Bengali served as the motivation for this work. Despite being spoken by millions worldwide, the Bengali language continues to lack robust tools for text summarization and domain adaptation, particularly when compared to more widely spoken languages. This research contributes to the advancement of NLP for Bengali by promoting improved usability and accessibility in linguistic information processing.

For Bengali text summarization, the two LLM models were trained on the Bengali split of the XLSUM dataset, where both models were trained on different hyperparameters and numbers of epochs. Following this, evaluation metrics were determined for the two models using ROUGE, BLEU and BERTScore, which highlighted the strengths and weaknesses of the two models. The mT5 model outperformed the Flan-T5 model across most evaluation metrics, achieving ROUGE-1, ROUGE-2, and ROUGE-L scores of 0.21, 0.09, and 0.20, respectively, alongside a BLEU score of 0.17 and a BERTScore of 0.72. These metrics highlight the effectiveness with which mT5 performs on complex Bengali text summarization tasks, which can be ascribed to its multilingual pretraining on a variety of datasets, allowing it to achieve competitive results, particularly in resource-constrained settings. Table 16 compares the performance of various Bengali text summarization models based on ROUGE and BLEU metrics from past notable research publications. The comparison highlights the improvements achieved by the proposed domain-adaptive Flan-T5 model, which outperforms other approaches in domain adaptation tasks, achieving superior scores across all metrics.

**Table 16:** Comparison of existing Bangla text summarization works with the proposed approach.

Study	Model/Approach	Dataset	ROUGE-1	ROUGE-2	ROUGE-L	BLEU	Observations
[5]	Seq2Seq with Attention	BANSData (19,096 samples)	0.30	–	0.31	0.30	Challenges with long inputs; issues with repetition and factual inaccuracies.
[3]	Hybrid Pointer Generator Network	BANSData and BANS-133k (133,148 samples)	0.67	0.42	0.41	–	Improved accuracy; reduced repetition; stable on large datasets.
[4]	Ranking-Based Approach	XLSUM Sum Bengali (10,126), Bangla Text Summarization (80,148)	0.45	0.18	0.41	0.02	Enhanced summary accuracy by combining model outputs.
[38]	Chart-to-Text Summarization	Bengal ChartSumm (4,100 chart images)	0.0678	0.0016	0.0920	0.2779	Introduced a new dataset for chart-to-text summarization in Bengali; explored the potential of mT5 and BanglaT5 models.
**This work**	Domain-Adaptive Flan-T5	Custom Dataset (21,512)	0.67	0.57	0.67	0.46	Demonstrated superior performance in domain adaptation tasks.

Additional insights into the models’ domain-specific adaptability were obtained through domain adaptation tests conducted under both fine-tuning and zero-shot configurations. The performance of the state, international, and sports categories was evaluated using ROUGE, BLEU, and BERTScore metrics, following careful dataset selection. In the fine-tuning setting, Flan-T5 outperformed in terms of ROUGE and BERTScore, demonstrating strong generalization and semantic alignment capabilities within the state domain, with a ROUGE score of 0.79, a BLEU score of 0.13, and a BERTScore of 0.86. Conversely, mT5 exhibited superior n-gram matching accuracy, as reflected by its higher BLEU scores. In the zero-shot setting, where models were tested on previously unseen domain-specific datasets without additional training, both models achieved competitive results. Notably, the international domain yielded the highest performance in the zero-shot setting, with Flan-T5 attaining a ROUGE score of 0.67, a BLEU score of 0.47, and a BERTScore of 0.89. Fig 11 illustrates the comparative evaluation metrics of mT5 and Flan-T5 across the three domains under both settings. These findings underscore the robust zero-shot generalization capability of Flan-T5, particularly in linguistically diverse environments.

**Fig 11:**
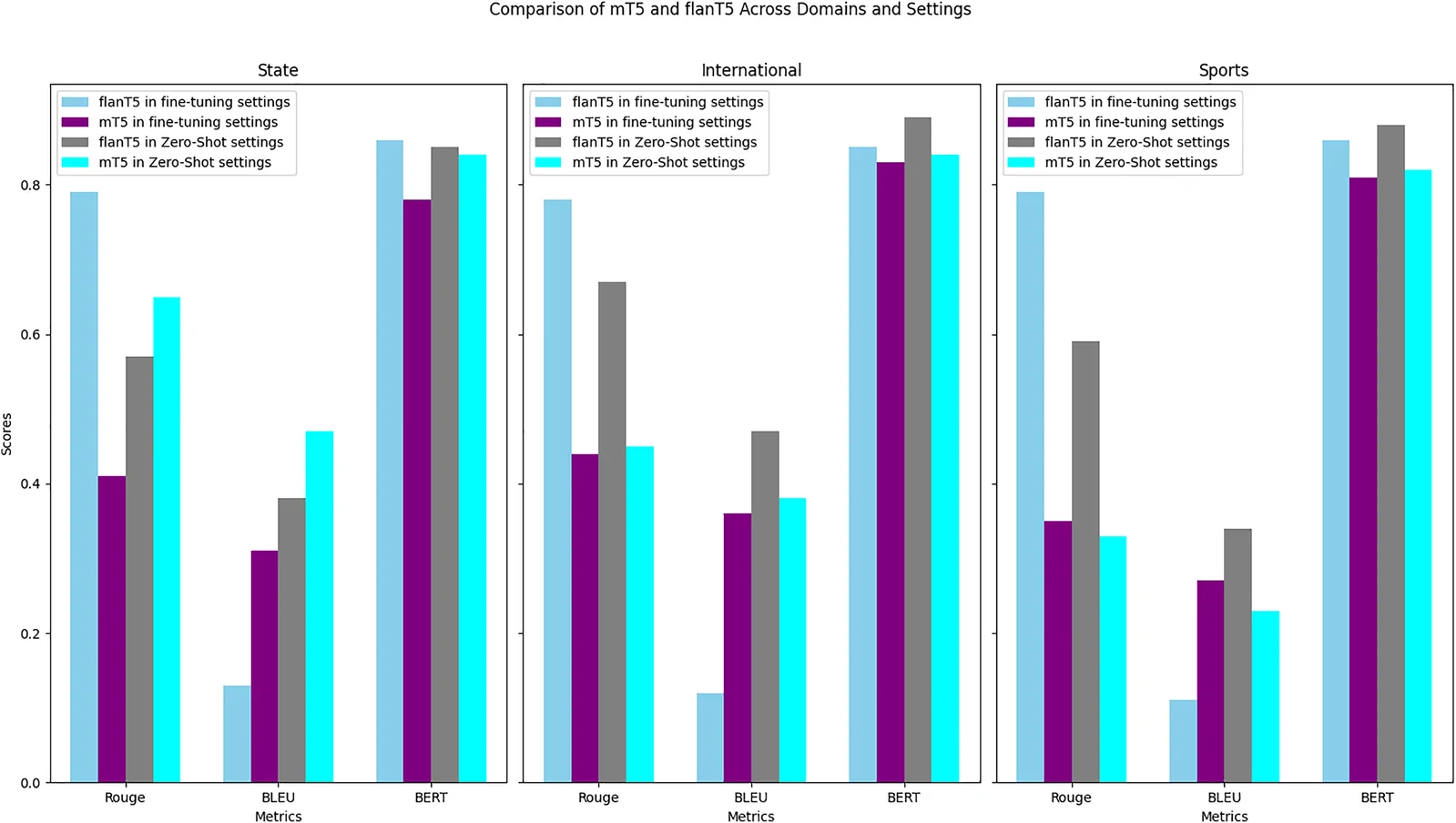
Comparison of evaluation parameters of mT5 and Flan-T5 models across three domains in different settings.

To enhance domain-specific performance without retraining the entire model, adapter modules were inserted between transformer layers of the Flan-T5 architecture. These lightweight modules enabled efficient fine-tuning, updating only 0.89% of total parameters (688K out of 77.6M). This approach allowed the model to adapt to target domains (State, Sports, and International) while preserving general language knowledge from pre-training. External evaluation was conducted alongside evaluation metrics for assessing the quality of generated summaries. As the last step after achieving all the training and evaluation parameter results, a user-friendly web application was developed using the Flask framework of Python. This application serves as an accessible interface for Bengali text summarization, where on the left, users input Bengali text into the box, and upon clicking the “Summarize” button, the summary is generated and displayed in the “Summary” section on the right. The user-friendly design of the interface makes it possible for people without technical knowledge to quickly and simply create insightful summaries, which makes it appropriate for a variety of uses, i.e., document analysis, educational content, and news summarizing. Through the integration of advanced LLMs and domain adaptation techniques, the research contributes to the development of accurate and practical tools for Bengali text summarization.

## Conclusion and future works

This research explored the application of various large language models for Bangla text summarization, among which the Flan-T5 and mT5 models demonstrated the capability to generate coherent summaries. These models were initially trained on the XLSum dataset. We have created our custom dataset, which contains 21,512 rows. This custom dataset was later categorized across seven different categories, of which the international and state categories had the highest frequency. This process was done as categorizing creates domains for Domain Adaptation to be applied. The models were fine-tuned on three domains, i.e., state, international and sports. The models were evaluated under fine-tuning and zero-shot configurations, using ROUGE, BLEU, and BERTScore metrics to assess performance. The Flan-T5 model exhibited superior performance in the fine-tuning setting, particularly within the state domain, achieving ROUGE and BERTScore values of 0.79 and 0.86, respectively. In contrast, the mT5 model demonstrated strong performance in zero-shot scenarios, indicating its capacity to generalize to unseen data. In particular, the international domain performed the best in the zero-shot setting with Flan-T5, achieving a ROUGE score of 0.67, a BLEU score of 0.47, and a BERTS score of 0.89.

The findings were supported by an external evaluation, where both Flan-T5 and mT5 achieved high scores for fluency, adequacy, and factual consistency across all domains. This reinforces the real-world applicability and linguistic reliability of the generated Bangla summaries.

To improve the efficiency of the model and reduce computational cost, we implemented an adapter-augmented architecture on the Flan-T5 model. The adapter modules enabled lightweight domain adaptation by training only 0.89% (≈688K) of the total 77.6M parameters, while keeping the base model frozen. This approach significantly improved the fine-tuning efficiency, allowing the model to specialize across domains with minimal additional training cost and memory usage.

### Limitations

The custom Bengali news-based dataset constructed in this work is limited to seven categories, with three (state, international, and sports) utilized for domain adaptation. This limitation affects the models’ ability to generalize across a wider range of domains. The evaluation process mainly depends on automated metrics such as ROUGE, BLEU, and BERTScore, which may not adequately reflect the cultural and linguistic subtleties of Bengali text. Training and fine-tuning large language models, mT5 and Flan-T5, demand considerable computational resources, which may not be practical in environments with limited resources. Although zero-shot evaluations showed competitive results, their performance varied by domain, suggesting that the models might have difficulty with specific nuances in unfamiliar contexts. Despite the external evaluation providing valuable qualitative insights, the sample size (28 participants) was relatively small and can be expanded in future work for broader validation. In the case of adapter tuning, which substantially reduced the number of trainable parameters, the performance gains were modest in some domains. Exploration of adapter placement strategies and multi-adapter fusion may yield stronger improvements.

### Future works

In the future, the dataset can be expanded to cover additional domains such as healthcare, technology, and legal documents, as well as to a larger multilingual scale, to improve the model’s adaptability and application range. Data augmentation strategies can be employed. Evaluating state-of-the-art models, such as GPT-4 and BongLLaMA, may provide improved benchmarks and understanding of Bengali text summarization capabilities. The user interface of the proposed system can be enhanced to support real-time feedback, and adopting a more intuitive design would improve accessibility and user experience. Exploring adaptation techniques such as partial and universal domain adaptation and implementing hybrid LLMs could lead to a more thorough understanding of model performance across various settings. Future studies can involve a larger and more diverse set of human evaluators to capture broader linguistic and cultural variations in human judgment of Bangla summaries. Future research can investigate domain-specific and cross-domain adapter fusion techniques to improve generalization and transfer learning across Bengali text domains. Incorporating parameter-efficient fine-tuning (PEFT) methods, such as LoRA or prefix-tuning, alongside adapters could enhance adaptability and reduce training overhead. Discourse-aware or hierarchical explainability approaches can be investigated to provide deeper interpretive insights beyond token-level analysis.

## Supporting information

S1 File English translations of non-English text appearing in the manuscript. The file provides English translations of the Bengali and other non-English text presented in Table 7 and Figures 2, 5, 8, 9, and 10.
(DOCX)

S1 Data Dataset files. (ZIP)
