# Supplementary material for: Domain-adaptive Bengali text summarization: Harnessing transformer-based large language models in closed-set contexts
Source: PLoS One. 2026 Oct 5;21(10):e0357890. doi: 10.1371/journal.pone.0357890 (PMC13638053; doi:10.1371/journal.pone.0357890)
Supplement: Supplementary file 1 [file pone.0357890.s003.pdf]

Response for Reviewers

We would like to thank the reviewers for their comments. In the following, we will present our responses to the comments together with a summary of the corresponding changes in the revised manuscript in Yellow Highlights. All page numbers mentioned below are referred in the new revised manuscript in Red Fonts. Following what the reviewer suggested, we have done the followings:

Reviewer 1:

1. **Comment:** "Although a custom dataset of ~21k entries was created, it is still relatively small compared to large-scale multilingual corpora."

   Response: Thank you for the valuable comment. We acknowledge that our dataset is smaller than large-scale multilingual corpora. However, our intention was to construct a focused, domain-specific dataset representing Bangladeshi news sources for effective domain adaptation and contextual relevance. Expanding the dataset to a larger multilingual scale remains an important direction for our future work. (Page 26)

2. **Comment:** "The dataset covers only seven domains, with experiments focusing mainly on state, international, and sports, leaving out others like education, economy, and technology."

   Response: We acknowledge that the experiments were primarily focused on three domains (state, international, and sports) due to data balance and computational constraints. However, the remaining domains (education, economy, and technology) are included in the dataset and will be explored in future work to provide a more comprehensive evaluation across all categories. (page 26)

3. **Comment:** "The study relies primarily on ROUGE, BLEU, and BERTScore, which have known shortcomings in capturing semantic quality and factual correctness."

   Response: Following what the reviewer suggested, the models were further evaluated using additional metrics, including distinct-1, distinct-2, and self-BLEU scores in Table 9. Evaluation of generated summaries using Distinct-1/2 and Self-BLEU scores." (Page 20)

4. **Comment:** "Human evaluation is not reported, limiting insight into real-world usability and fluency of the generated summaries."

   Response: Following what the reviewer suggested, we have added a detailed human evaluation section (see External Evaluation Section) to complement the automated metrics. This section describes our evaluation setup with 28 native Bangla-speaking participants, assessing fluency, adequacy, and factual consistency using a five-point Likert scale. The inclusion of these results provides deeper insight into the real-world usability and linguistic quality of the generated summaries. (Page 20)

5. **Comment:** "The approach assumes that source and target domains share the same label space (closed set). This limits applicability in more realistic open-domain scenarios where unseen categories often occur."

   Response: Thank you for the insightful comment. We acknowledge that our approach assumes a shared label space between the source and target domains, representing a closed-set adaptation scenario. To clarify this design choice and its rationale, we have added a dedicated subsection titled "Domain Adaptation in Closed-Set Context," which explicitly describes the shared label space and explains that this setup was chosen to focus on learning invariant features while avoiding additional complexity from label mismatches. (Page 12)

6. **Comment:** "Comparisons are restricted to Flan-T5 and mT5. Other strong multilingual LLMs (e.g., GPT, LLaMA-based models, or Bengali-specific LMs like Bangla-BERT) are mentioned in literature review but not directly benchmarked against."

   Response: Following the reviewer's suggestion, we have applied two Bengali-specific models—Bangla-BERT and BanglaT5—in our revised experiments. These models provide stronger native-language baselines, allowing for a more comprehensive comparison with the multilingual LLMs. The updated results and evaluation parameters are presented in Table 7. Evaluation parameters of BanglaBERT and BanglaT5 models for comparative analysis in Bengali text summarization. (Page 17)

7. **Comment:** "Some Key related papers missing: Sanodiya, Rakesh Kumar, et al. "A kernelized unified framework for domain adaptation." IEEE Access 7 (2019): 181381-181395. Paul, Dipanjyoti, et al. "Online summarization of microblog data: An aid in handling disaster situations." IEEE Transactions on Computational Social Systems 11.3 (2024): 4029-4039)."

   Response: Following the reviewer's suggestion, we have the literature reviews of the suggessted articles in [31] and [32]. (Page 6)

8. **Comment:** "the dataset distribution, requiring resampling. This imbalance may bias model performance and does not fully reflect natural domain variations."

   Response: Thank you for the insightful observation. We acknowledge the dataset imbalance and its potential influence on model performance. To address this, we have clarified in the revised manuscript that resampling was applied to balance the data distribution across domains, with recognition that this may slightly limit natural domain variation. Details are added in Domain Adaptation in Closed-Set Context Section. (Page 12)

9. **Comment:** "Tools like LIME, SHAP, and BertViz provide token-level insights but do not necessarily capture higher-level discourse structures in Bengali, potentially limiting the interpretability of the results."

   Response: We have expanded the Explainability and Interpretability Analysis section to include detailed discussions of LIME, SHAP, and BertViz outputs for Bengali text summarization. Additionally, we have acknowledged that while these tools provide valuable

token-level insights, they are limited in capturing higher-level discourse structures, which we plan to explore in future work. (Page 23)

Reviewer 2:

1. **Comment:** "The authors mainly fine-tunes existing pretrained LLMs (Flan-T5, mT5) on an existing dataset (XLSum) and a relatively small custom dataset. They do not introduce any new architecture."

   Response: While our work primarily builds upon pretrained LLMs, we have now incorporated an adapter-based parameter-efficient fine-tuning (PEFT) strategy using LoRA to introduce a lightweight yet effective architectural extension. This approach enables domain-specific adaptation with minimal computational cost and provides a novel contribution beyond conventional fine-tuning. Details are added in Section Adapter-Based Fine-Tuning for Parameter-Efficient Domain Adaptation. (page 13)

2. **Comment:** "Key experimental details are omitted. You mention domain specific fine tuning, but how?."

   Response: Following the reviewer's suggestion, we have expanded the Training LLM models on Bengali Domain for Text Summarization section to include detailed steps of the domain-specific fine-tuning process, including corpus preparation, tokenizer training using SentencePiece, vocabulary merging with T5, and reinitialization of the Flan-T5 tokenizer for Bengali adaptation. (page 11)

3. **Comment:** "authors can provide the actual citation of 7, 8 9, 12,14 and etc references"

   Response: Thanks. Done. (page 27)

4. **Comment:** "the detailed description of ROUGE, BLEU, BERTScore are missing in the article."

   Response: Thanks. Added in Section: Evaluation Parameters for Domain Adaptation in different settings. (page 18)

5. **Comment:** "hyperparameters λ1 and λ2 regulate the domain adaptation losses' contributions" but how are they deciding these values not provided and also what is the range of these parameters."

   Response: The hyperparameters λ_1 and λ_2 were tuned and fixed using the Optuna tool to balance the contributions of the domain classification and domain confusion losses. Specifically, λ_1 and λ_2 were varied within the range [0.1, 1.0].

6. **Comment:** "Caption of table 1 seems inappropriate."

Response: Thanks. Caption of Table I has been changed to Summary of Related Studies on Bengali and Multilingual Text Summarization Models. (page 7)

7. **Comment:** “the analysis of figure 9 and figure 11 has not been given.”

   Response: Thanks. Added in Explainability and Interpretability Analysis Section. (page 23-24)
