# Supplementary material for: Domain-adaptive Bengali text summarization: Harnessing transformer-based large language models in closed-set contexts
Source: PLoS One. 2026 Oct 5;21(10):e0357890. doi: 10.1371/journal.pone.0357890 (PMC13638053; doi:10.1371/journal.pone.0357890)
Supplement: Supplementary file 2 [file pone.0357890.s005.pdf]

Response for Reviewers

We would like to thank the reviewers for their comments. In the following, we will present our responses to the comments together with a summary of the corresponding changes in the revised manuscript in Yellow Highlights. All page numbers mentioned below are referred in the new revised manuscript in Red Fonts. Following what the reviewer suggested, we have done the followings:

Reviewer 3:

1. **Comment:** “since ROUGE compares n-grams, how can one ensure that the generated abstracts are actually easy to read, sufficiently complex, and coherent?”

   Response: We respectfully agree that ROUGE alone cannot ensure that generated summaries are easy to read, sufficiently complex, and coherent, because it mainly evaluates n-gram overlap with reference summaries. In our study, we complemented ROUGE with BLEU, BERTScore, distinct-1, distinct-2, and self-BLEU to capture both lexical and semantic similarity, and more importantly, we conducted a human evaluation with 28 participants to assess the generated summaries in terms of fluency, adequacy, and factual consistency across domains. This human evaluation was included to verify whether the summaries are readable, coherent, and informative from a user perspective. See Table 9. Evaluation of generated summaries using Distinct-1/2 and Self-BLEU scores.” (Page 19)

2. **Comment:** “In addition, the initialization of the embeddings should be described in more detail.”

   Response: The model was initialized with pretrained weights from the Flan-T5 architecture and further adapted to Bengali using a custom tokenizer. The embedding layer of the pretrained Flan-T5 model was resized to match the Bengali tokenizer vocabulary. Newly introduced tokens were randomly initialized, while existing token embeddings retained their pretrained weights.

3. **Comment:** “The authors should also clearly specify which parameters were used in their experiments.”

   Response: Following what the reviewer suggested, we have added a concise hyperparameter table in Table 4: Hyperparameter settings used for Flan-T5 and mT5 models.” (Page 16)

4. **Comment:** “It would also be valuable to discuss other approaches to text modeling that could be considered (perhaps in the Related Work section). For example, graph-based approaches such as 'using complex networks to quantify consistency in the use of words' (jstat, 2012) and 'forma mentis networks quantify crucial differences in STEM perception between students and experts' (plos one, 2019) could be mentioned as other possible ideas to identify central sentences in texts. While these are only examples of graph techniques, it would be interesting to mention how graph based approaches could be combined with your approach.”

Response: Following what the reviewer suggested, we added "Antonio et al. [33] investigated graph-based text modeling approaches employing complex-network analysis of word usage consistency and forma mentis networks for text representation. The authors provided valuable perspectives for identifying important textual structures and central sentences. Stell et al. [34] forma mentis networks for neural summarization. Sentence or word centrality scores as auxiliary features, ranking signals, or pre-selection mechanisms are applied before abstractive summary generation. The authors proved that graph-based representations could be combined with the domain-adapted frameworks." (Page 6)

Reviewer 4:

1. **Comment:** "Most of the pipeline relies on fine-tuning existing pretrained models, and the adapter and LoRA extension are useful but incremental rather than a fundamentally new method."

   Response: We respectfully agree that the proposed framework builds on existing pretrained models and that the adapter/LoRA-based extension is incremental rather than a completely new model architecture. Our main contribution is therefore not claimed as the invention of a fundamentally new LLM method, but as a domain-adaptive Bengali summarization framework for a low-resource language, supported by a newly developed manually categorized Bengali news dataset, systematic domain-wise fine-tuning, ablation analysis, human evaluation, explainability analysis, and parameter-efficient adaptation. The novelty of this work is implementing a domain-adaptive Bengali text summarization framework using fine-tuned large language models (mT5 and Flan-T5) on a newly created, manually categorized dataset, demonstrating improved summarization performance in low-resource, domain-specific contexts. (page 3)

2. **Comment:** "The study is framed as domain adaptation, but the setting is limited to a closed-set scenario with only three overlapping categories. This makes the conclusions less generalizable to realistic open-domain or unseen-domain condition."

   Response: We agree that the current domain adaptation setting is limited to a closed-set scenario involving three selected domains: state, international, and sports. These domains were selected because they contained the largest number of samples and allowed a more balanced and reliable experimental comparison under our available computational resources. We acknowledge that this setting does not fully represent realistic open-domain or unseen-domain summarization conditions. In the revised manuscript, we have clarified this limitation and avoided overgeneralizing the findings beyond the evaluated domains. We also note that the remaining domains in our dataset, including education, economy, technology, and entertainment, provide a useful basis for future work, where the framework can be extended to broader multi-domain, cross-domain, and unseen-domain adaptation settings. (page 27)

3. **Comment:** "The custom dataset is valuable, but experiments use only a subset of the seven annotated domains, and class imbalance is handled through resampling, which may distort natural data distributions and inflate apparent gains."

   Response: We agree that although the custom dataset contains seven annotated domains, our experiments used only three domains, namely state, international, and sports, because these categories had the largest number of samples and allowed a more stable comparison under the available computational resources. We also acknowledge that resampling may change the natural class distribution and may affect the interpretation of performance gains. In the revised manuscript, we have clarified that resampling was used only to reduce severe imbalance during training and that the results should be interpreted within this controlled closed-domain setting rather than as evidence of full open-domain generalization. We have also added weighted loss functions in the revised manuscript.

4. **Comment:** "Evaluation is improved compared with the earlier version. However, it still lacks stronger evidence of robustness. There is no clear statistical significance analysis, no detailed error analysis, and limited discussion of hallucinations or factual failures beyond small-scale human scoring."

   Response: In the revised manuscript, we have added a statistical significance analysis using paired t-tests on sample-level ROUGE-1 scores, showing that the improvements of the domain-adapted models over the baseline are significant across the state, international, and sports domains, with all p-values below 0.001. We have also expanded the discussion of robustness by acknowledging remaining limitations, including possible hallucinations, factual inconsistencies, and domain-specific errors that may not be fully captured by ROUGE-based evaluation or small-scale human scoring. See Section: Robustness and Statistical Significance Analysis. (page 23)

5. **Comment:** "The human evaluation remains relatively small, with only 28 participants, so the qualitative conclusions should be treated cautiously."

   Response: We agree that the human evaluation with 28 participants provides useful qualitative feedback but remains relatively small, and therefore the findings should be interpreted cautiously. In the revised manuscript, we have clarified that the external evaluation is intended to complement the automatic metrics rather than provide definitive evidence of human-perceived quality.

6. **Comment:** "Reproducibility is better than before, but some experimental choices still need sharper reporting, especially full split protocol, domain-wise sample counts after resampling, variance across runs, and clearer justification of hyperparameter sensitivity."

   Response: In the revised manuscript, we have expanded the experimental setup by specifying the full train/validation/test split protocol, domain-wise sample counts before and after resampling, and the final hyperparameter settings selected using Optuna. Details in Table 4: Hyperparameter settings used for Flan-T5 and mT5 models." (Page 16)

7. **Comment:** "LIME, SHAP, and BertViz mainly provide token-level illustrations and do not validate whether the model captures discourse-level summarization behavior."

   Response: We have expanded the Explainability and Interpretability Analysis section to include detailed discussions of LIME, SHAP, and BertViz outputs for Bengali text summarization. Additionally, we have acknowledged that while these tools provide valuable token-level insights, they are limited in capturing higher-level discourse structures, which we plan to explore in future work. (Page 23)
