# Supplementary material for: Domain-adaptive Bengali text summarization: Harnessing transformer-based large language models in closed-set contexts
Source: PLoS One. 2026 Oct 5;21(10):e0357890. doi: 10.1371/journal.pone.0357890 (PMC13638053; doi:10.1371/journal.pone.0357890)
Supplement: Supplementary file 3 [file pone.0357890.s001.docx]

**Supplementary File S1**

English Translations of Non-English Text Appearing in the Manuscript

**Table 7 (Sample of generated summaries using the two models) — English translations**

Location in final manuscript: Table 7, page 17

| **Section** | **Content** |
| --- | --- |
| Article | Activists are calling for Coke and Pepsi to be banned in other regions of India as well. Businesspeople undertook this initiative to ensure the use of local products. The state’s two leading traders’ associations had proposed banning these two beverages. Consequently, Coca-Cola and Pepsi were banned in the state of Tamil Nadu beginning Wednesday.  The organizations say that soft-drink companies use large quantities of water from rivers, causing severe difficulties for farmers when irrigating their land. The shortage of irrigation water becomes particularly acute during periods of drought. More than one million shopkeepers across the state are expected to comply with the ban.  Last month, Tamil Nadu witnessed widespread protests against the prohibition of the traditional bull-taming event known as “Jallikattu.” Following these protests, the state’s two leading trade organizations—the Federation of Tamil Nadu Traders Associations (FTNTA) and the Tamil Nadu Traders Association—proposed banning Pepsi and Coca-Cola. During the protests, many people argued that banning “Jallikattu” amounted to disrespecting local traditions and culture.  “We began our campaign against soft drinks several months ago. However, when we started protesting against the ban on ‘Jallikattu,’ our campaign against soft drinks also took a different form,” said FTNTA President T. Vellaiyan in an interview with the BBC Tamil Service.  “Beverages such as Pepsi and Coca-Cola are not good for your health because they contain various chemicals and excessive amounts of sugar. We are promoting Indian soft drinks, and we will also continue our efforts to increase the sale of fruit juices,” said businessman T. Vellaiyan.  Considering the development of local businesses and farmers, the associations also urged supermarkets, restaurants, and hotels to comply with the ban. Pepsi and Coca-Cola have not yet commented on the ban. |
| Reference Summary | Businesspeople in the southern Indian state of Tamil Nadu have announced a ban on the sale of Coca-Cola and Pepsi there. |
| Text Summarization (mT5) | Businesspeople have banned Coca-Cola and Pepsi in the Indian state of Tamil Nadu. These two beverages use large quantities of water from rivers, causing severe difficulties for farmers when irrigating their land. |
| Text Summarization (Flan-T5) | Businesspeople undertook this initiative to ban other uses in India. |
| Domain Adaptation (mT5) | Businesspeople have banned Coca-Cola and Pepsi in the Indian state of Tamil Nadu. |
| Domain Adaptation (Flan-T5) | Activists are calling for Coke and Pepsi to be banned in other regions of India as well. Businesspeople undertook this initiative to ensure the use of local products. The state’s two leading traders’ associations had proposed banning these two beverages. Consequently, it was banned in the state of Tamil Nadu beginning Wednesday. |

**Figure 2 (Sample entries from the custom Bengali news summarization dataset.)**

**Location:** Figure 2, page 10 of the final manuscript

| **Category** | **Title** | **Text** | **Summary** |
| --- | --- | --- | --- |
| Entertainment | ‘Hason Raja Festival’ in Sydney on 9 December | In memory of Hason Raja, the ‘Hason Raja Festival’ is being held in Sydney, Australia. On 9 December, at Sydney’s Banks… | In memory of Hason Raja, the ‘Hason Raja Festival’ is being held in Sydney, Australia. |
| Entertainment | Bangla cinema returns to the Emirates; a film festival will be held | Bangladeshis have undertaken plans to arrange Bangla film releases, film festivals, and studio facilities in the Emirates… | Arrangements are being made in the Emirates for Bangla film releases, film festivals, and studios… |
| Education | Study for life, not life for study | It is now the season of gentle autumn breezes. When one goes for a walk, colorful leaves can be seen lying beneath one’s feet. The weather is quite calm… | It is now the season of gentle autumn breezes. When one goes for a walk, colorful leaves can be seen lying beneath one’s feet… |
| State | Two separate rallies in New York protesting the attack on poet Radhapada Roy | Expatriate Bangladeshis held a rally in New York, United States, to protest the attack on poet Tarapada Roy. Lalon… | Expatriates held a rally in New York, United States, to protest the attack on poet Tarapada Roy… |
| International | Expatriates offer prayers at Surau Baitul Mokarram in Malaysia on the night of Shab-e-Qadr | Devout expatriates are offering prayers at Surau Baitul Mokarram on the holy night of Shab-e-Qadr. On Saturday night, at Surau Baitu… | Devout expatriates offered prayers at Surau Baitul Mokarram on the holy night of Shab-e-Qadr… |
| International | Tribute to the Language Martyrs in Dubai | On the occasion of Martyrs’ Day and International Mother Language Day, tribute was paid to the Language Martyrs in Dubai, the commercial city of the United Arab Emirates… | On the occasion of Martyrs’ Day and International Mother Language Day, in the commercial city of the United Arab Emirates… |

**Figure 5 (Web interface of the proposed Bengali text summarization system.)**

**Location:** Figure 5, page 15 of the final manuscript

| Input Text | On Thursday afternoon, more than five hundred people, including local journalists, lawyers, athletes, members of different professions, and representatives of social organizations, participated in the program held at the city’s main intersection.  Speaking at the rally, the participants said:  “Recently, a group of people presenting themselves on various social-media platforms as journalists and human-rights activists have been mocking the Liberation War and making unsolicited and indecent remarks about prominent political figures, established members of civil society, and members of their families.”  They further stated:  “As part of this, some individuals have been using YouTube and Facebook to spread fabricated and unwarranted propaganda about Mirza Fakhrul Islam Alamgir, one of the country’s leading political figures, and his family. This is not beneficial to society at all.” |
| --- | --- |
| Generated Summary | Recently in Bangladesh, a group of people presenting themselves on various social-media platforms as journalists and human-rights activists have been mocking the Liberation War and making unsolicited and indecent remarks about prominent political figures, established members of civil society, and members of their families. As part of this, some individuals… |

**Figure 8 (LIME Visualization of Token Importance for Flan-T5 Summary Prediction in the Sports Domain.)**

**Location:** Figure 8, page 24 of the final manuscript

| **Bengali word** | **English translation** |
| --- | --- |
| মধ্যপ্রাচ্যের | Middle Eastern / of the Middle East |
| দেশ | country |
| ওমানের | Oman’s / of Oman |
| জাতীয় | national |
| লীগের | league’s / of the league |

**Figure 9 (SHAP Explanation of Token-Level Contributions in Flan-T5 Summary Prediction for the Sports Domain.)**

**Location:** Figure 9, page 24 of the final manuscript

| Model output | Bangladesh Cricket Club has qualified to compete in the “Senior D” division of the national league of Oman, a Middle Eastern country. The country’s… |
| --- | --- |
| Input text | Bangladesh Cricket Club has qualified to compete in the “Senior D” division of the national league of Oman, a Middle Eastern country. The club, which represents Bangladesh and is composed of expatriate players, achieved this distinction by finishing as the runner-up in the group stage of the “E” division of the Senior Division T20 Cricket League, organized by the country’s cricket board.  In this eight-team group, a team of expatriate Indian players became the champion with 12 points. Bangladesh Cricket Club finished as the runner-up with 10 points and was promoted to the “Senior D” division along with the champion team.  In the decisive match played on Friday at a cricket stadium in Muscat, Bangladesh Cricket Club defeated Friends Players, a team composed of expatriate Indian and Pakistani players, by 25 runs. After winning the toss, Bangladesh elected to bat first. Owing to strong performances from its batters, the team scored 171 runs in the allotted 20 overs. Saiful Islam was the highest scorer for Bangladesh, making 59 runs from 37 balls. Other players also contributed useful runs. Among the opposing bowlers, Mohammad Abrar took three wickets for 37 runs, while other bowlers also claimed wickets.  In reply, Friends Players, chasing a target of 172 runs, scored 146 runs for the loss of seven wickets in the allotted 20 overs. Teja Shetty scored 33 runs, while the other batters also contributed. For Bangladesh Cricket Club, one bowler took two wickets for 22 runs, while Sunny, Mizan, Saiful, and Alam took one wicket each.  Saiful Islam of Bangladesh Cricket Club was named Player of the Match for scoring 59 runs and contributing with the ball. He was also selected as Player of the Tournament for scoring the highest number of runs.  Bangladesh Cricket Club began its journey in 2021 through the initiative of several sports-loving expatriates. Within a short period, the club became known among athletes in Oman and subsequently achieved consistent success. It began in the “F” division, was promoted to the “E” division last year, and has now qualified for the “Senior D” division. The club also has a women’s cricket team. The expatriate Bangladeshi community and club officials are highly encouraged by this latest achievement.  Club President Ashraful Rahman said:  “We never imagined that we would be able to achieve this success so quickly. However, we had confidence that, considering the hard work and dedication of our players, we would not have to wait long to reach our goal.”  Club Secretary Sheikh Fahad thanked the Oman Cricket Board, sponsors, players, and expatriate Bangladeshis and said:  “Our responsibilities have now increased considerably. We are making larger plans to move the team forward. We hope that sponsors and members of the Bangladeshi community will continue to support and assist us in this regard.” |

**Figure 10 (BertViz Explanation of Token-Level Contributions in Flan-T5 Summary Prediction for the Sports Domain.)**

**Location:** Figure 10, page 24 of the final manuscript

| **Bengali token** | **English translation** |
| --- | --- |
| মধ্যপ্রাচ্যের | Middle Eastern / of the Middle East |
| দেশ | country |
| ওমানের | Oman’s / of Oman |
| জাতীয় | national |
| লীগের | of the league |
| সিনিয়র | Senior |
| ডি | D |
| বিভাগে | in the division |
| খেলার | to play / to compete |
| যোগ্যতা | qualification / eligibility |
| অর্জন | achieve |
| করেছে | has achieved |
| বাংলাদেশ | Bangladesh |
| ক্রিকেট | Cricket |
| ক্লাব | Club |
| দেশটির | of the country |
| ক্রিকেট | cricket |
| বোর্ডের | of the board |
| ব্যবস্থাপনায় | under the management of |
| সিনিয়র | Senior |
| ডিভিশন | Division |
| টি২০ | T20 |
| ক্রিকেট | Cricket |
| লিগ | League |
