# Supplementary material for: Domain-adaptive Bengali text summarization: Harnessing transformer-based large language models in closed-set contexts
Source: PLoS One. 2026 Oct 5;21(10):e0357890. doi: 10.1371/journal.pone.0357890 (PMC13638053; doi:10.1371/journal.pone.0357890)
Supplement: Supplementary file 4 [file pone.0357890.s004.docx]

**Reviewer Comments: Dr Rimsha Anam**

**I have carefully read the manuscript and believe that further improvements are required for it to meet the standards of PLOS ONE. The following comments need to be addressed**:

1. Some sections of the manuscript still contain long sentences and repetitive wording especially in the introduction and related work sections. A careful language revision would improve readability.

2. Figures presenting explainability results (LIME, SHAP, BertViz) should include clearer captions and interpretation explaining what the highlighted tokens indicate.

3. The manuscript should report training hyperparameters more clearly, including:

- learning rate
- batch size
- number of epochs
- hardware configuration

4. Some tables could be improved by adding standard deviation or variance especially if results are averaged over multiple runs.

5. The discussion section could better highlight practical applications of Bengali domain-adaptive summarization systems.
