# Supplementary material for: Domain-adaptive Bengali text summarization: Harnessing transformer-based large language models in closed-set contexts
Source: PLoS One. 2026 Oct 5;21(10):e0357890. doi: 10.1371/journal.pone.0357890 (PMC13638053; doi:10.1371/journal.pone.0357890)
Supplement: Peer review history

# Peer review: Decision Letter 0

**DOI:** 10.1371/journal.pone.0357890.r001

**Authors:** Hossein Hassani

12 Sep 2025

Dear Dr. Khan,

plosone@plos.org. When you're ready to submit your revision, log on to https://www.editorialmanager.com/pone/ and select the 'Submissions Needing Revision' folder to locate your manuscript file.

- A rebuttal letter that responds to each point raised by the academic editor and reviewer(s). You should upload this letter as a separate file labeled 'Response to Reviewers'.
- A marked-up copy of your manuscript that highlights changes made to the original version. You should upload this as a separate file labeled 'Revised Manuscript with Track Changes'.
- An unmarked version of your revised paper without tracked changes. You should upload this as a separate file labeled 'Manuscript'.

If you would like to make changes to your financial disclosure, please include your updated statement in your cover letter. Guidelines for resubmitting your figure files are available below the reviewer comments at the end of this letter.

If applicable, we recommend that you deposit your laboratory protocols in protocols.io to enhance the reproducibility of your results. Protocols.io assigns your protocol its own identifier (DOI) so that it can be cited independently in the future. For instructions see: https://journals.plos.org/plosone/s/submission-guidelines#loc-laboratory-protocols. Additionally, PLOS ONE offers an option for publishing peer-reviewed Lab Protocol articles, which describe protocols hosted on protocols.io. Read more information on sharing protocols at https://plos.org/protocols?utm_medium=editorial-email&utm_source=authorletters&utm_campaign=protocols.

We look forward to receiving your revised manuscript.

Kind regards,

Hossein Hassani, PhD

Academic Editor

PLOS ONE

Journal Requirements:

When submitting your revision, we need you to address these additional requirements.

1. Please ensure that your manuscript meets PLOS ONE's style requirements, including those for file naming. The PLOS ONE style templates can be found at

https://journals.plos.org/plosone/s/file?id=wjVg/PLOSOne_formatting_sample_main_body.pdf and

https://journals.plos.org/plosone/s/file?id=ba62/PLOSOne_formatting_sample_title_authors_affiliations.pdf

2. In your Methods section, please include additional information about your dataset and ensure that you have included a statement specifying whether the collection and analysis method complied with the terms and conditions for the source of the data.

3. Please note that PLOS One has specific guidelines on code sharing for submissions in which author-generated code underpins the findings in the manuscript. In these cases, we expect all author-generated code to be made available without restrictions upon publication of the work. Please review our guidelines at https://journals.plos.org/plosone/s/materials-and-software-sharing#loc-sharing-code and ensure that your code is shared in a way that follows best practice and facilitates reproducibility and reuse.

4. We note that your Data Availability Statement is currently as follows: [All relevant data are within the manuscript and its Supporting Information files.]

Please confirm at this time whether or not your submission contains all raw data required to replicate the results of your study. Authors must share the “minimal data set” for their submission. PLOS defines the minimal data set to consist of the data required to replicate all study findings reported in the article, as well as related metadata and methods (https://journals.plos.org/plosone/s/data-availability#loc-minimal-data-set-definition).

For example, authors should submit the following data:

- The values behind the means, standard deviations and other measures reported;

- The values used to build graphs;

- The points extracted from images for analysis.

Authors do not need to submit their entire data set if only a portion of the data was used in the reported study.

If your submission does not contain these data, please either upload them as Supporting Information files or deposit them to a stable, public repository and provide us with the relevant URLs, DOIs, or accession numbers. For a list of recommended repositories, please see https://journals.plos.org/plosone/s/recommended-repositories.

If there are ethical or legal restrictions on sharing a de-identified data set, please explain them in detail (e.g., data contain potentially sensitive information, data are owned by a third-party organization, etc.) and who has imposed them (e.g., an ethics committee). Please also provide contact information for a data access committee, ethics committee, or other institutional body to which data requests may be sent. If data are owned by a third party, please indicate how others may request data access.

5. If the reviewer comments include a recommendation to cite specific previously published works, please review and evaluate these publications to determine whether they are relevant and should be cited. There is no requirement to cite these works unless the editor has indicated otherwise.

[Note: HTML markup is below. Please do not edit.]

Reviewers' comments:

Reviewer's Responses to Questions

**Comments to the Author**

1. Is the manuscript technically sound, and do the data support the conclusions?

Reviewer #1: Yes

Reviewer #2: Partly

**********

2. Has the statistical analysis been performed appropriately and rigorously?

Reviewer #1: Yes

Reviewer #2: No

**********

3. Have the authors made all data underlying the findings in their manuscript fully available?

The PLOS Data policy

Reviewer #1: Yes

Reviewer #2: No

**********

4. Is the manuscript presented in an intelligible fashion and written in standard English?

Reviewer #1: Yes

Reviewer #2: Yes

**********

Reviewer #1: The study investigates domain-adaptive Bengali text summarization using transformer-based large language models (LLMs), specifically Flan-T5 and mT5. The work addresses the scarcity of effective summarization systems for Bengali, a low-resource language, by training models on both general datasets (XLSUM) and a newly created, manually annotated dataset covering seven domains (state, international, sports, entertainment, economy, education, and technology).

• Although a custom dataset of ~21k entries was created, it is still relatively small compared to large-scale multilingual corpora.

• The dataset covers only seven domains, with experiments focusing mainly on state, international, and sports, leaving out others like education, economy, and technology.

• The study relies primarily on ROUGE, BLEU, and BERTScore, which have known shortcomings in capturing semantic quality and factual correctness.

• Human evaluation is not reported, limiting insight into real-world usability and fluency of the generated summaries.

• The approach assumes that source and target domains share the same label space (closed set).

• This limits applicability in more realistic open-domain scenarios where unseen categories often occur.

• Comparisons are restricted to Flan-T5 and mT5. Other strong multilingual LLMs (e.g., GPT, LLaMA-based models, or Bengali-specific LMs like Bangla-BERT) are mentioned in literature review but not directly benchmarked against.

• Some Key related papers missing: Sanodiya, Rakesh Kumar, et al. "A kernelized unified framework for domain adaptation." IEEE Access 7 (2019): 181381-181395.

Paul, Dipanjyoti, et al. "Online summarization of microblog data: An aid in handling disaster situations." IEEE Transactions on Computational Social Systems 11.3 (2024): 4029-4039.

• the dataset distribution, requiring resampling. This imbalance may bias model performance and does not fully reflect natural domain variations

• Tools like LIME, SHAP, and BertViz provide token-level insights but do not necessarily capture higher-level discourse structures in Bengali, potentially limiting the interpretability of the results.

Reviewer #2: The article Domain-Adaptive Bengali Text Summarization: Harnessing Transformer-Based Large Language Models in Closed-Set Contexts attempt to address Bengali text Summarization that is an important problem in the current research communities. In this article authors has shown some effort in applying existing LLMs to Bengali text summarization but it lacks novelty, rigorous evaluation. We have witness very few works related to Bengali however this task has been already seen with major languages like english and some sort of Bengali language also.

The author try to focus on leveraging LLMs like FLAN-T5 and mT5 and incorporating domain adaptation for the Bengali language. The creation of datasets of around 20000 instances in 7 domains has a useful step for this bengali language.

I have some of the observation in the current form of the article:

�The authors mainly fine-tunes existing pretrained LLMs (Flan-T5, mT5) on an existing dataset (XLSum) and a relatively small custom dataset. They do not introduce any new architecture.

�Key experimental details are omitted. You mention domain specific fine tuning, but how?

�authors can provide the actual citation of 7, 8 9, 12,14 and etc references.

�the detailed description of ROUGE, BLEU, BERTScore are missing in the article.

�"hyperparameters λ1 and λ2 regulate the domain adaptation losses’ contributions" but how are they deciding these values not provided and also what is the range of these parameters.

�Caption of table 1 seems inappropriate.

�the analysis of figure 9 and figure 11 has not been given.

The study lacks crucial experimental rigor, including fine-tuning details, statistical testing. There is no mention of fine-tuning processes, statistical significance testing, random seeding, multiple runs. Such omissions severely limit the validity and reproducibility of your results. Implementing these methodological practices is necessary to enhance the scientific reliability of your findings.

**********

what does this mean?). If published, this will include your full peer review and any attached files.

If you choose “no”, your identity will remain anonymous but your review may still be made public.

**Do you want your identity to be public for this peer review?**  For information about this choice, including consent withdrawal, please see our Privacy Policy

Reviewer #1: No

Reviewer #2: No

**********

[NOTE: If reviewer comments were submitted as an attachment file, they will be attached to this email and accessible via the submission site. Please log into your account, locate the manuscript record, and check for the action link "View Attachments". If this link does not appear, there are no attachment files.]

While revising your submission, please upload your figure files to the Preflight Analysis and Conversion Engine (PACE) digital diagnostic tool, https://pacev2.apexcovantage.com/. PACE helps ensure that figures meet PLOS requirements. To use PACE, you must first register as a user. Registration is free. Then, login and navigate to the UPLOAD tab, where you will find detailed instructions on how to use the tool. If you encounter any issues or have any questions when using PACE, please email PLOS at figures@plos.org. Please note that Supporting Information files do not need this step.

# Author comment: Author response to Decision Letter 1

**DOI:** 10.1371/journal.pone.0357890.r002

20 Nov 2025

Response for Reviewers

We would like to thank the reviewers for their comments. In the following, we will present our responses to the comments together with a summary of the corresponding changes in the revised manuscript in Yellow Highlights. All page numbers mentioned below are referred in the new revised manuscript in Red Fonts. Following what the reviewer suggested, we have done the followings:

Reviewer 1:

1. Comment: “Although a custom dataset of ~21k entries was created, it is still relatively small compared to large-scale multilingual corpora.”

Response: Thank you for the valuable comment. We acknowledge that our dataset is smaller than large-scale multilingual corpora. However, our intention was to construct a focused, domain-specific dataset representing Bangladeshi news sources for effective domain adaptation and contextual relevance. Expanding the dataset to a larger multilingual scale remains an important direction for our future work. (Page 26)

2. Comment: “The dataset covers only seven domains, with experiments focusing mainly on state, international, and sports, leaving out others like education, economy, and technology.”

Response: We acknowledge that the experiments were primarily focused on three domains (state, international, and sports) due to data balance and computational constraints. However, the remaining domains (education, economy, and technology) are included in the dataset and will be explored in future work to provide a more comprehensive evaluation across all categories. (page 26)

3. Comment: “The study relies primarily on ROUGE, BLEU, and BERTScore, which have known shortcomings in capturing semantic quality and factual correctness.”

Response: Following what the reviewer suggested, the models were further evaluated using additional metrics, including distinct-1, distinct-2, and self-BLEU scores in Table 9. Evaluation of generated summaries using Distinct-1/2 and Self-BLEU scores.” (Page 20)

4. Comment: “Human evaluation is not reported, limiting insight into real-world usability and fluency of the generated summaries.”

Response: Following what the reviewer suggested, we have added a detailed human evaluation section (see External Evaluation Section) to complement the automated metrics. This section describes our evaluation setup with 28 native Bangla-speaking participants, assessing fluency, adequacy, and factual consistency using a five-point Likert scale. The inclusion of these results provides deeper insight into the real-world usability and linguistic quality of the generated summaries. (Page 20)

5. Comment: “The approach assumes that source and target domains share the same label space (closed set). This limits applicability in more realistic open-domain scenarios where unseen categories often occur.”

Response: Thank you for the insightful comment. We acknowledge that our approach assumes a shared label space between the source and target domains, representing a closed-set adaptation scenario. To clarify this design choice and its rationale, we have added a dedicated subsection titled “Domain Adaptation in Closed-Set Context,” which explicitly describes the shared label space and explains that this setup was chosen to focus on learning invariant features while avoiding additional complexity from label mismatches. (Page 12)

6. Comment: “Comparisons are restricted to Flan-T5 and mT5. Other strong multilingual LLMs (e.g., GPT, LLaMA-based models, or Bengali-specific LMs like Bangla-BERT) are mentioned in literature review but not directly benchmarked against.”

Response: Following the reviewer’s suggestion, we have applied two Bengali-specific models—Bangla-BERT and BanglaT5—in our revised experiments. These models provide stronger native-language baselines, allowing for a more comprehensive comparison with the multilingual LLMs. The updated results and evaluation parameters are presented in Table 7. Evaluation parameters of BanglaBERT and BanglaT5 models for comparative analysis in Bengali text summarization. (Page 17)

7. Comment: “Some Key related papers missing: Sanodiya, Rakesh Kumar, et al. "A kernelized unified framework for domain adaptation." IEEE Access 7 (2019): 181381-181395. Paul, Dipanjyoti, et al. "Online summarization of microblog data: An aid in handling disaster situations." IEEE Transactions on Computational Social Systems 11.3 (2024): 4029-4039).”

Response: Following the reviewer’s suggestion, we have the literature reviews of the suggessted articles in [31] and [32]. (Page 6)

8. Comment: “the dataset distribution, requiring resampling. This imbalance may bias model performance and does not fully reflect natural domain variations.”

Response: Thank you for the insightful observation. We acknowledge the dataset imbalance and its potential influence on model performance. To address this, we have clarified in the revised manuscript that resampling was applied to balance the data distribution across domains, with recognition that this may slightly limit natural domain variation. Details are added in Domain Adaptation in Closed-Set Context Section. (Page 12)

9. Comment: “Tools like LIME, SHAP, and BertViz provide token-level insights but do not necessarily capture higher-level discourse structures in Bengali, potentially limiting the interpretability of the results.”

Response: We have expanded the Explainability and Interpretability Analysis section to include detailed discussions of LIME, SHAP, and BertViz outputs for Bengali text summarization. Additionally, we have acknowledged that while these tools provide valuable token-level insights, they are limited in capturing higher-level discourse structures, which we plan to explore in future work. (Page 23)

Reviewer 2:

1. Comment: “The authors mainly fine-tunes existing pretrained LLMs (Flan-T5, mT5) on an existing dataset (XLSum) and a relatively small custom dataset. They do not introduce any new architecture.”

Response: While our work primarily builds upon pretrained LLMs, we have now incorporated an adapter-based parameter-efficient fine-tuning (PEFT) strategy using LoRA to introduce a lightweight yet effective architectural extension. This approach enables domain-specific adaptation with minimal computational cost and provides a novel contribution beyond conventional fine-tuning. Details are added in Section Adapter-Based Fine-Tuning for Parameter-Efficient Domain Adaptation. (page 13)

2. Comment: “Key experimental details are omitted. You mention domain specific fine tuning, but how?.”

Response: Following the reviewer’s suggestion, we have expanded the Training LLM models on Bengali Domain for Text Summarization section to include detailed steps of the domain-specific fine-tuning process, including corpus preparation, tokenizer training using SentencePiece, vocabulary merging with T5, and reinitialization of the Flan-T5 tokenizer for Bengali adaptation. (page 11)

3. Comment: “authors can provide the actual citation of 7, 8 9, 12,14 and etc references”

Response: Thanks. Done. (page 27)

4. Comment: “the detailed description of ROUGE, BLEU, BERTScore are missing in the article.”

Response: Thanks. Added in Section: Evaluation Parameters for Domain Adaptation in different settings. (page 18)

5. Comment: “hyperparameters λ1 and λ2 regulate the domain adaptation losses’ contributions" but how are they deciding these values not provided and also what is the range of these parameters.”

Response: The hyperparameters λ₁ and λ₂ were tuned and fixed using the Optuna tool to balance the contributions of the domain classification and domain confusion losses. Specifically, λ₁ and λ₂ were varied within the range [0.1, 1.0].

6. Comment: “Caption of table 1 seems inappropriate.”

Response: Thanks. Caption of Table I has been changed to Summary of Related Studies on Bengali and Multilingual Text Summarization Models. (page 7)

7. Comment: “the analysis of figure 9 and figure 11 has not been given.”

Response: Thanks. Added in Explainability and Interpretability Analysis Section. (page 23-24)

Attachment Submitted filename: PONE-D-25-33130R1_Response.pdf

# Peer review: Decision Letter 1

**DOI:** 10.1371/journal.pone.0357890.r003

**Authors:** Hossein Hassani

6 Apr 2026

Dear Dr. Khan,

Please submit your revised manuscript by May 21 2026 11:59PM. If you will need more time than this to complete your revisions, please reply to this message or contact the journal office at plosone@plos.org. When you're ready to submit your revision, log on to https://www.editorialmanager.com/pone/ and select the 'Submissions Needing Revision' folder to locate your manuscript file.

- A letter that responds to each point raised by the academic editor and reviewer(s). You should upload this letter as a separate file labeled 'Response to Reviewers'.
- A marked-up copy of your manuscript that highlights changes made to the original version. You should upload this as a separate file labeled 'Revised Manuscript with Track Changes'.
- An unmarked version of your revised paper without tracked changes. You should upload this as a separate file labeled 'Manuscript'.

If you would like to make changes to your financial disclosure, please include your updated statement in your cover letter. Guidelines for resubmitting your figure files are available below the reviewer comments at the end of this letter.

If applicable, we recommend that you deposit your laboratory protocols in protocols.io to enhance the reproducibility of your results. Protocols.io assigns your protocol its own identifier (DOI) so that it can be cited independently in the future. For instructions see: https://journals.plos.org/plosone/s/submission-guidelines#loc-laboratory-protocols. Additionally, PLOS ONE offers an option for publishing peer-reviewed Lab Protocol articles, which describe protocols hosted on protocols.io. Read more information on sharing protocols at https://plos.org/protocols?utm_medium=editorial-email&utm_source=authorletters&utm_campaign=protocols.

As the corresponding author, your ORCID iD is verified in the submission system and will appear in the published article. PLOS supports the use of ORCID, and we encourage all coauthors to register for an ORCID iD and use it as well. Please encourage your coauthors to verify their ORCID iD within the submission system before final acceptance, as unverified ORCID iDs will not appear in the published article. *Only*  the individual author can complete the verification step; PLOS staff *cannot*  verify ORCID iDs on behalf of authors.

We look forward to receiving your revised manuscript.

Kind regards,

Hossein Hassani, PhD

Academic Editor

PLOS One

Journal Requirements:

If the reviewer comments include a recommendation to cite specific previously published works, please review and evaluate these publications to determine whether they are relevant and should be cited. There is no requirement to cite these works unless the editor has indicated otherwise.

[Note: HTML markup is below. Please do not edit.]

Reviewers' comments:

Reviewer's Responses to Questions

**Comments to the Author**

Reviewer #3: (No Response)

Reviewer #4: (No Response)

**********

2. Is the manuscript technically sound, and do the data support the conclusions?

Reviewer #3: Partly

Reviewer #4: Yes

**********

3. Has the statistical analysis been performed appropriately and rigorously?

Reviewer #3: I Don't Know

Reviewer #4: Yes

**********

4. Have the authors made all data underlying the findings in their manuscript fully available?

The PLOS Data policy

Reviewer #3: Yes

Reviewer #4: Yes

**********

5. Is the manuscript presented in an intelligible fashion and written in standard English?

Reviewer #3: Yes

Reviewer #4: Yes

**********

Reviewer #3: This paper proposes an interesting approach for domain-adaptive Bengali Text Summarization. My main concerns regarding this manuscript are: since ROUGE compares n-grams, how can one ensure that the generated abstracts are actually easy to read, sufficiently complex, and coherent? In addition, the initialization of the embeddings should be described in more detail. The authors should also clearly specify which parameters were used in their experiments. It would also be valuable to discuss other approaches to text modeling that could be considered (perhaps in the Related Work section). For example, graph-based approaches such as 'using complex networks to quantify consistency in the use of words' (jstat, 2012) and 'forma mentis networks quantify crucial differences in STEM perception between students and experts' (plos one, 2019) could be mentioned as other possible ideas to identify central sentences in texts. While these are only examples of graph techniques, it would be interesting to mention how graph based approaches could be combined with your approach.

Reviewer #4: Here is a concise technical review report of the paper. The manuscript proposes Bengali domain-adaptive abstractive summarization using mT5 and Flan-T5, trained on XLSUM and a custom 21,512-instance Bengali news dataset, and evaluated across three domains: state, international, and sports. The revised version also adds Bengali-specific baselines, human evaluation, and a LoRA-based parameter-efficient tuning variant. The topic is relevant, especially for low-resource language summarization, and the paper’s main strength is its attempt to combine domain adaptation, custom data creation, and qualitative analysis within a single framework.

Drawbacks:

1. Most of the pipeline relies on fine-tuning existing pretrained models, and the adapter and LoRA extension are useful but incremental rather than a fundamentally new method.

2. The study is framed as domain adaptation, but the setting is limited to a closed-set scenario with only three overlapping categories. This makes the conclusions less generalizable to realistic open-domain or unseen-domain conditions.

3. The custom dataset is valuable, but experiments use only a subset of the seven annotated domains, and class imbalance is handled through resampling, which may distort natural data distributions and inflate apparent gains.

4. Evaluation is improved compared with the earlier version. However, it still lacks stronger evidence of robustness. There is no clear statistical significance analysis, no detailed error analysis, and limited discussion of hallucinations or factual failures beyond small-scale human scoring.

5. The human evaluation remains relatively small, with only 28 participants, so the qualitative conclusions should be treated cautiously.

6. Reproducibility is better than before, but some experimental choices still need sharper reporting, especially full split protocol, domain-wise sample counts after resampling, variance across runs, and clearer justification of hyperparameter sensitivity.

7. LIME, SHAP, and BertViz mainly provide token-level illustrations and do not validate whether the model captures discourse-level summarization behavior.

**********

what does this mean?). If published, this will include your full peer review and any attached files.

If you choose “no”, your identity will remain anonymous but your review may still be made public.

**Do you want your identity to be public for this peer review?**  For information about this choice, including consent withdrawal, please see our Privacy Policy

Reviewer #3: No

Reviewer #4: No

**********

[NOTE: If reviewer comments were submitted as an attachment file, they will be attached to this email and accessible via the submission site. Please log into your account, locate the manuscript record, and check for the action link "View Attachments". If this link does not appear, there are no attachment files.]

To ensure your figures meet our technical requirements, please review our figure guidelines: https://journals.plos.org/plosone/s/figures

You may also use PLOS’s free figure tool, NAAS, to help you prepare publication quality figures: https://journals.plos.org/plosone/s/figures#loc-tools-for-figure-preparation.

NAAS will assess whether your figures meet our technical requirements by comparing each figure against our figure specifications.

Attachment Submitted filename: Reviewer Comments.docx

# Author comment: Author response to Decision Letter 2

**DOI:** 10.1371/journal.pone.0357890.r004

2 May 2026

Response for Reviewers

We would like to thank the reviewers for their comments. In the following, we will present our responses to the comments together with a summary of the corresponding changes in the revised manuscript in Yellow Highlights. All page numbers mentioned below are referred in the new revised manuscript in Red Fonts. Following what the reviewer suggested, we have done the followings:

Reviewer 3:

1. Comment: “since ROUGE compares n-grams, how can one ensure that the generated abstracts are actually easy to read, sufficiently complex, and coherent?”

Response: We respectfully agree that ROUGE alone cannot ensure that generated summaries are easy to read, sufficiently complex, and coherent, because it mainly evaluates n-gram overlap with reference summaries. In our study, we complemented ROUGE with BLEU, BERTScore, distinct-1, distinct-2, and self-BLEU to capture both lexical and semantic similarity, and more importantly, we conducted a human evaluation with 28 participants to assess the generated summaries in terms of fluency, adequacy, and factual consistency across domains. This human evaluation was included to verify whether the summaries are readable, coherent, and informative from a user perspective. See Table 9. Evaluation of generated summaries using Distinct-1/2 and Self-BLEU scores.” (Page 19)

2. Comment: “In addition, the initialization of the embeddings should be described in more detail.”

Response: The model was initialized with pretrained weights from the Flan-T5 architecture and further adapted to Bengali using a custom tokenizer. The embedding layer of the pretrained Flan-T5 model was resized to match the Bengali tokenizer vocabulary. Newly introduced tokens were randomly initialized, while existing token embeddings retained their pretrained weights.

3. Comment: “The authors should also clearly specify which parameters were used in their experiments.”

Response: Following what the reviewer suggested, we have added a concise hyperparameter table in Table 4: Hyperparameter settings used for Flan-T5 and mT5 models.” (Page 16)

4. Comment: “It would also be valuable to discuss other approaches to text modeling that could be considered (perhaps in the Related Work section). For example, graph-based approaches such as 'using complex networks to quantify consistency in the use of words' (jstat, 2012) and 'forma mentis networks quantify crucial differences in STEM perception between students and experts' (plos one, 2019) could be mentioned as other possible ideas to identify central sentences in texts. While these are only examples of graph techniques, it would be interesting to mention how graph based approaches could be combined with your approach.”

Response: Following what the reviewer suggested, we added “Antonio et al. [33] investigated graph-based text modeling approaches employing complex-network analysis of word usage consistency and forma mentis networks for text representation. The authors provided valuable perspectives for identifying important textual structures and central sentences. Stell et al. [34] forma mentis networks for neural summarization. Sentence or word centrality scores as auxiliary features, ranking signals, or pre-selection mechanisms are applied before abstractive summary generation. The authors proved that graph-based representations could be combined with the domain-adapted frameworks.” (Page 6)

Reviewer 4:

1. Comment: “Most of the pipeline relies on fine-tuning existing pretrained models, and the adapter and LoRA extension are useful but incremental rather than a fundamentally new method.”

Response: We respectfully agree that the proposed framework builds on existing pretrained models and that the adapter/LoRA-based extension is incremental rather than a completely new model architecture. Our main contribution is therefore not claimed as the invention of a fundamentally new LLM method, but as a domain-adaptive Bengali summarization framework for a low-resource language, supported by a newly developed manually categorized Bengali news dataset, systematic domain-wise fine-tuning, ablation analysis, human evaluation, explainability analysis, and parameter-efficient adaptation. The novelty of this work is implementing a domain-adaptive Bengali text summarization framework using fine-tuned large language models (mT5 and Flan-T5) on a newly created, manually categorized dataset, demonstrating improved summarization performance in low-resource, domain-specific contexts. (page 3)

2. Comment: “The study is framed as domain adaptation, but the setting is limited to a closed-set scenario with only three overlapping categories. This makes the conclusions less generalizable to realistic open-domain or unseen-domain condition.”

Response: We agree that the current domain adaptation setting is limited to a closed-set scenario involving three selected domains: state, international, and sports. These domains were selected because they contained the largest number of samples and allowed a more balanced and reliable experimental comparison under our available computational resources. We acknowledge that this setting does not fully represent realistic open-domain or unseen-domain summarization conditions. In the revised manuscript, we have clarified this limitation and avoided overgeneralizing the findings beyond the evaluated domains. We also note that the remaining domains in our dataset, including education, economy, technology, and entertainment, provide a useful basis for future work, where the framework can be extended to broader multi-domain, cross-domain, and unseen-domain adaptation settings. (page 27)

3. Comment: “The custom dataset is valuable, but experiments use only a subset of the seven annotated domains, and class imbalance is handled through resampling, which may distort natural data distributions and inflate apparent gains.”

Response: We agree that although the custom dataset contains seven annotated domains, our experiments used only three domains, namely state, international, and sports, because these categories had the largest number of samples and allowed a more stable comparison under the available computational resources. We also acknowledge that resampling may change the natural class distribution and may affect the interpretation of performance gains. In the revised manuscript, we have clarified that resampling was used only to reduce severe imbalance during training and that the results should be interpreted within this controlled closed-domain setting rather than as evidence of full open-domain generalization. We have also added weighted loss functions in the revised manuscript.

4. Comment: “Evaluation is improved compared with the earlier version. However, it still lacks stronger evidence of robustness. There is no clear statistical significance analysis, no detailed error analysis, and limited discussion of hallucinations or factual failures beyond small-scale human scoring.”

Response: In the revised manuscript, we have added a statistical significance analysis using paired t-tests on sample-level ROUGE-1 scores, showing that the improvements of the domain-adapted models over the baseline are significant across the state, international, and sports domains, with all p-values below 0.001. We have also expanded the discussion of robustness by acknowledging remaining limitations, including possible hallucinations, factual inconsistencies, and domain-specific errors that may not be fully captured by ROUGE-based evaluation or small-scale human scoring. See Section: Robustness and Statistical Significance Analysis. (page 23)

5. Comment: “The human evaluation remains relatively small, with only 28 participants, so the qualitative conclusions should be treated cautiously.”

Response: We agree that the human evaluation with 28 participants provides useful qualitative feedback but remains relatively small, and therefore the findings should be interpreted cautiously. In the revised manuscript, we have clarified that the external evaluation is intended to complement the automatic metrics rather than provide definitive evidence of human-perceived quality.

6. Comment: “Reproducibility is better than before, but some experimental choices still need sharper reporting, especially full split protocol, domain-wise sample counts after resampling, variance across runs, and clearer justification of hyperparameter sensitivity.”

Response: In the revised manuscript, we have expanded the experimental setup by specifying the full train/validation/test split protocol, domain-wise sample counts before and after resampling, and the final hyperparameter settings selected using Optuna. Details in Table 4: Hyperparameter settings used for Flan-T5 and mT5 models.” (Page 16)

7. Comment: “LIME, SHAP, and BertViz mainly provide token-level illustrations and do not validate whether the model captures discourse-level summarization behavior.”

Response: We have expanded the Explainability and Interpretability Analysis section to include detailed discussions of LIME, SHAP, and BertViz outputs for Bengali text summarization. Additionally, we have acknowledged that while these tools provide valuable token-level insights, they are limited in capturing higher-level discourse structures, which we plan to explore in future work. (Page 23)

Attachment Submitted filename: Plos_Summarization__response_v2.pdf

# Peer review: Decision Letter 2

**DOI:** 10.1371/journal.pone.0357890.r005

**Authors:** Shuai Liu

24 Aug 2026

Domain-Adaptive Bengali Text Summarization: Harnessing Transformer-Based Large Language Models in Closed-Set Contexts

PONE-D-25-33130R2

Dear Dr. Khan,

We’re pleased to inform you that your manuscript has been judged scientifically suitable for publication and will be formally accepted for publication once it meets all outstanding technical requirements.

Within one week, you’ll receive an e-mail detailing the required amendments. When these have been addressed, you’ll receive a formal acceptance letter and your manuscript will be scheduled for publication.

An invoice will be generated when your article is formally accepted. Please note, if your institution has a publishing partnership with PLOS and your article meets the relevant criteria, all or part of your publication costs will be covered. Please make sure your user information is up-to-date by logging into Editorial Manager at Editorial Manager® and clicking the ‘Update My Information' link at the top of the page. For questions related to billing, please contact billing support.

If your institution or institutions have a press office, please notify them about your upcoming paper to help maximize its impact. If they’ll be preparing press materials, please inform our press team as soon as possible -- no later than 48 hours after receiving the formal acceptance. Your manuscript will remain under strict press embargo until 2 pm Eastern Time on the date of publication. For more information, please contact onepress@plos.org.

Kind regards,

Shuai Liu

Academic Editor

PLOS One

Additional Editor Comments (optional):

Reviewers' comments:

Reviewer's Responses to Questions

**Comments to the Author**

Reviewer #4: All comments have been addressed

**********

2. Is the manuscript technically sound, and do the data support the conclusions?

Reviewer #4: Yes

**********

3. Has the statistical analysis been performed appropriately and rigorously?

Reviewer #4: Yes

**********

4. Have the authors made all data underlying the findings in their manuscript fully available?

The PLOS Data policy

Reviewer #4: Yes

**********

5. Is the manuscript presented in an intelligible fashion and written in standard English?

Reviewer #4: Yes

**********

Reviewer #4: Thanks for the authors for considering reviewers' comments and recommendations. The paper can be accepted.

**********

what does this mean?). If published, this will include your full peer review and any attached files.

If you choose “no”, your identity will remain anonymous but your review may still be made public.

**Do you want your identity to be public for this peer review?**  For information about this choice, including consent withdrawal, please see our Privacy Policy

Reviewer #4: No

**********

# Editor report: Acceptance letter

**DOI:** 10.1371/journal.pone.0357890.r006

**Authors:** Shuai Liu

PONE-D-25-33130R2

PLOS One

Dear Dr. Khan,

I'm pleased to inform you that your manuscript has been deemed suitable for publication in PLOS One. Congratulations! Your manuscript is now being handed over to our production team.

At this stage, our production department will prepare your paper for publication. This includes ensuring the following:

* All references, tables, and figures are properly cited

* All relevant supporting information is included in the manuscript submission,

* There are no issues that prevent the paper from being properly typeset

You will receive further instructions from the production team, including instructions on how to review your proof when it is ready. Please keep in mind that we are working through a large volume of accepted articles, so please give us a few days to review your paper and let you know the next and final steps.

Lastly, if your institution or institutions have a press office, please let them know about your upcoming paper now to help maximize its impact. If they'll be preparing press materials, please inform our press team within the next 48 hours. Your manuscript will remain under strict press embargo until 2 pm Eastern Time on the date of publication. For more information, please contact onepress@plos.org.

You will receive an invoice from PLOS for your publication fee after your manuscript has reached the completed accept phase. If you receive an email requesting payment before acceptance or for any other service, this may be a phishing scheme. Learn how to identify phishing emails and protect your accounts at https://explore.plos.org/phishing.

If we can help with anything else, please email us at customercare@plos.org.

Thank you for submitting your work to PLOS One and supporting open access.

Kind regards,

PLOS One Editorial Office Staff

on behalf of

Dr. Shuai Liu

Academic Editor

PLOS One
